# Free-base porphyrin polymer for bifunctional electrochemical water splitting†

**DOI:** 10.1039/d2sc01250b

**Published:** 2022-07-04

**Authors:** Yulu Ge, Zhenhua Lyu, Mariana Marcos-Hernández, Dino Villagrán

**Affiliations:** Department of Chemistry and Biochemistry, The University of Texas at El Paso El Paso TX 79968 USA dino@utep.edu

## Abstract

Water splitting is considered a promising approach for renewable and sustainable energy conversion. The development of water splitting electrocatalysts that have low-cost, long-lifetime, and high-performance is an important area of research for the sustainable generation of hydrogen and oxygen gas. Here, we report a metal-free porphyrin-based two-dimensional crystalline covalent organic polymer obtained from the condensation of terephthaloyl chloride and 5,10,15,20-tetrakis(4-aminophenyl) porphyrin which is stabilized by an extensive hydrogen bonding network. This material exhibits bifunctional electrocatalytic performance towards water splitting with onset overpotentials, *η*, of 36 mV and 110 mV for HER (in 0.5 M H_2_SO_4_) and OER (in 1.0 M KOH), respectively. The as-synthesized material is also able to perform water splitting in neutral phosphate buffer saline solution, with 294 mV for HER and 520 mV for OER, respectively. Characterized by electrochemical impedance spectroscopy (EIS) and chronoamperometry, the as-synthesized material also shows enhanced conductivity and stability compared to its molecular counterpart. Inserting a non-redox active zinc metal center in the porphyrin unit leads to a decrease in electrochemical activity towards both HER and OER, suggesting the four-nitrogen porphyrin core is the active site. The high performance of this metal-free material towards water splitting provides a sustainable alternative to the use of scarce and expensive metal electrocatalysts in energy conversion for industrial applications.

## Abstract 2

Water splitting is considered a promising approach for renewable and sustainable energy conversion.

## Graphical abstract

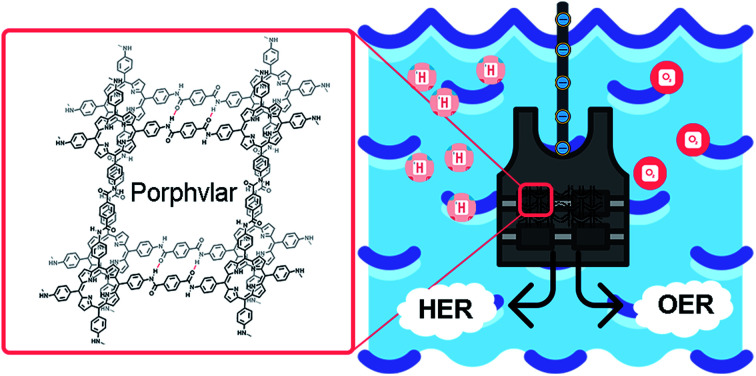

## Introduction

Global energy consumption increased drastically within the past 20 years and it is expected to continue to grow over the next several decades.^1^ The rapid depletion of non-renewable resources and their related combustion issues require the development of alternative energy sources and technologies with zero carbon footprint.^2^ Hydrogen is an ideal energy carrier due to its high energy density compared with conventional fossil fuels and because water is its only combustion by-product.^3,4^ Currently, the production of hydrogen gas in industry is dependent on methane steam reforming which renders its production unsustainable and carbon positive.^5^ Theoretically, water can be an abundant source of hydrogen if hydrogen and oxygen gases can be produced through electrolysis. Water splitting requires a four-electron redox process in addition to concomitant proton transfer, and high thermodynamic demands (1.23 eV at pH 7.0).^6^ Current research efforts are focused on using renewable energy sources to induce water splitting directly^7,8^ or indirectly^9,10^ with minimal environmental effects. While electrochemical water splitting is effective and promising,^11^ commercial electrolyzers require robust and efficient catalysts to accomplish the hydrogen and the oxygen evolution reactions, HER and OER, respectively.^6,12^

Current electrocatalysts that meet these demands are based on scarce and expensive transition metals (*i.e.*, Pt elements for HER and RuO_2_/IrO_2_ for OER).^13–15^ Thus, the use of single earth-abundant materials as bifunctional catalysts for both HER and OER is desired for sustainable and economic feasibility.

Porphyrins are organic macrocycles that have conjugated aromatic rings and characteristic intense colors.^16^ Porphyrin-based catalysts for water splitting have been exclusively focused on metallated complexes.^17–26^ The generation of high-valent metal-oxo intermediates is the key step to form molecular oxygen, where the porphyrin scaffold serves only as a molecular frame to facilitate the multi-electron transfer process, and a metal center is responsible for the catalytic activity.^23,27,28^ Similar mechanisms are invoked in HER which have metal-hydride intermediates as proton transfer carriers.^18–20^

However, it has been shown that the catalytic activity of porphyrin complexes towards hydrogen generation does not necessarily require a metal center.^29,30^ The N–H groups and N-lone pairs in the core of free-base porphyrins can also act as active sites for hydrogen generation under acidic conditions.^31^ Electronic tuning at the *meso*-position by electron donating or withdrawing groups can yield porphyrin complexes with varied basicity that lead to distinct redox reactivity.^32,33^ Our previous work shows that a metal-free porphyrin with perfluorinated *meso*-substituted groups can electrocatalyze hydrogen generation in acid with a potential of −1.31 V (*vs.* Fc/Fc^+^) and 90% faradaic efficiency.^30^ However, molecular level electrocatalysts suffer from stability issues, low current densities, high cost, and non-recyclability for industrial utilization.^34^ Therefore, developing low-cost heterogeneous electrocatalysts that possess high reactivity and stability towards water splitting could be a promising strategy for hydrogen and oxygen production.

**Scheme 1:**
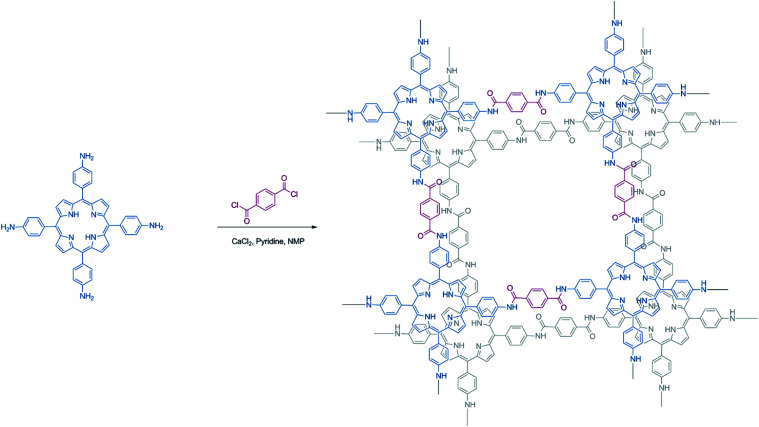
Synthesis procedure of Porphvlar.

Poly(*p*-phenylene terephthalamide) (PPTA) fibers are a type of ultra-strong synthetic polymer with a high tensile strength-to-weight ratio.^35^ The amide linkages form hydrogen bonds between the polymer chains which act like “hydrogen bond locks”,^36^ making it a material for bullet-proof body armors.^37^ Incorporating units of free-base porphyrins into PPTA networks can result in two-dimensional (2D) porphyrin-based polymers with ordered columnar π-arrays. Porphyrin moieties can enhance aromatic stacking interactions and add charge transport properties, used in electrocatalytic applications. However, the use of metal-free porphyrin-based polymer as bifunctional electrocatalysts directly for both HER and OER is still underexplored.

In this work, we present the synthesis of a metal-free porphyrin based crystalline 2D organic polymer, Porphvlar, obtained from the condensation of terephthaloyl chloride and 5,10,15,20-tetrakis(4-aminophenyl porphyrin, namely H_2_TAPP), (Scheme 1), which is an effective bifunctional electrocatalyst for the OER and the HER in pH dependent and pH neutral solutions. The electrochemical response of this material is explored under oxidation and reduction conditions in order to study its catalytic activity, charge transfer, and stability.

## Results and discussion

**Fig. 1:**
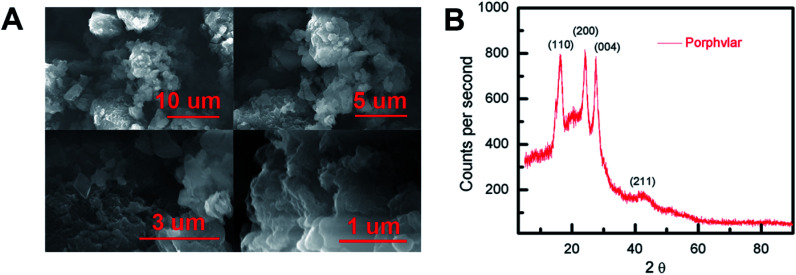
(A) Scanning electron microscopy (SEM) imaging of Porphvlar in various scales, and (B) pXRD pattern.

Scanning electron microscopy (SEM) images were obtained to evaluate the morphology of the synthesized Porphvlar polymer in different magnifications. Fig. 1A shows that the Porphvlar powder exhibits a flake-like morphology. The flakes are stacked in layers of a couple of nanometers in thickness. This is in contrast to the rod-like topology of PPTA threads.^38^ Both energy-dispersive X-ray spectroscopy (EDX) (Fig. S1†) and high-resolution XPS spectra (Fig. S6†) show that only C, N, and O elements exist throughout the Porphvlar structure without the presence of any metal atoms that could have been incorporated through the synthetic processes. Inductively coupled plasma-optical emission spectrometry (ICP-OES) was performed to more exhaustively study the elemental composition of the samples. A trace amount of Sn can be detected at the parts per million level (328 ppm) in Porphvlar samples. SnO is presumed to be the contaminant that is likely generated during the syntheses of the Porphvlar precursor, H_2_TAPP.

The powder X-ray diffraction (p-XRD) pattern in Fig. 1B shows that the sample is microcrystalline. The p-XRD pattern of Porphvlar closely resembles the crystalline pattern of commercial PPTA. The diffraction peaks observed at 2*θ* = 24.1°, 27.5°, 28.4° and 42.4° correspond to the (1 1 0), (2 0 0), (0 0 4) and (2 1 1) planes, respectively, which are also present in PPTA fibers.^39^ The broad peak centered at around 2*θ* = 20° is attributed to π–π stacking between the 2D layers, where the porphyrin units form an AA type eclipsed stacking.^40^

**Fig. 2:**
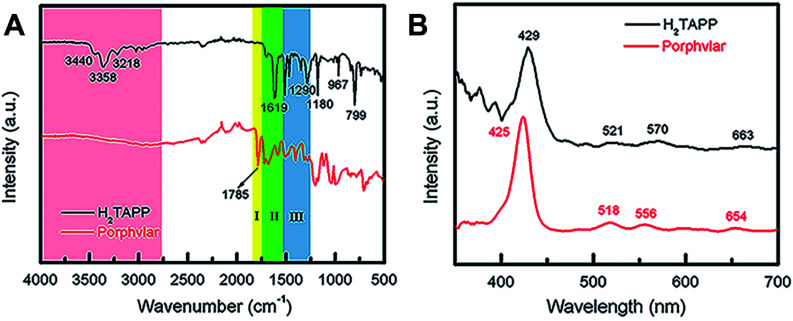
Spectroscopic characterization of Porphvlar. (A) FT-IR spectra of Porphvlar and molecular porphyrin (H_2_TAPP). (B) Comparison UV-vis spectra of Porphvlar and H_2_TAPP.

The FT-IR and UV-vis spectra of Porphvlar were compared to those obtained from the molecular porphyrin unit, H_2_TAPP. Fig. 2A shows the FT-IR spectra for both the porphyrin unit (top) and Porphvlar (bottom) materials. Porphvlar exhibits a band at 1785 cm^−1^ assigned to the amide I (carbonyl stretch, yellow region), the amide II band (N–H bend) appears around 1500–1700 cm^−1^ (green region), and the amide III band is observed in the blue region. These bands indicate the formation of the amide group in the Porphvlar structure and are missing in the H_2_TAPP spectrum. In addition, the *γ*-(N–H) pyrrole out-of-plane stretch is observed at 799 cm^−1^ in H_2_TAPP and shifts to ∼1000 cm^−1^ in Porphvlar. Two primary amines *ν*-(N–H) stretching bands at 3440 and 3356 cm^−1^ and one sp^2^*ν*-(C–H) stretching peak (3218 cm^−1^) are visible in H_2_TAPP, and those appear as a broad band around 3000 cm^−1^ in Porphvlar (pink region) due to hydrogen bonding between the polymer layers. Fig. 2B shows the UV-vis spectra of H_2_TAPP and Porphvlar. H_2_TAPP has the indicative spectrum of a free-base porphyrin with a Soret band at 429 nm along with its corresponding Q-bands between 521 to 663 nm. Porphvlar shares a similar spectrum where the Soret and Q bands are blue-shifted by about 4 nm. This indicates that the porphyrin unit is incorporated into the Porphvlar network.

The OER electrocatalytic activity evaluation of the resulting Porphvlar was performed in 1.0 M KOH aqueous solution and 1.0 M phosphate buffer saline solution (PBS) with carbon paper as the conductive support electrode. The carbon black/carbon paper blank electrode has a negligible current increase. In KOH solution, Porphvlar exhibits a current increase at 1.63 V (*η*_1_ = 400 mV) where a sharp increase is observed. The molecular porphyrin, H_2_TAPP, has a similar onset potential where the catalytic current is observed but with a lower current response (Fig. 2A, blue line). These onset overpotentials are lower than those from reported metallated porphyrin composite materials (rGO/(Ni^2+^/THPP/Co^2+^/THPP)_*n*_: 1.49 V *vs.* RHE)^41^ and other metal-based OER catalysts (FeNi LDH/GO: 1.439 V *vs.* RHE)^42^ and metal-free carbon/graphene-based multifunctional electrocatalysts (2D-N, S doped graphitic sheets: 1.49 V *vs.* RHE)^43^ in heterogeneous systems.

**Fig. 3:**
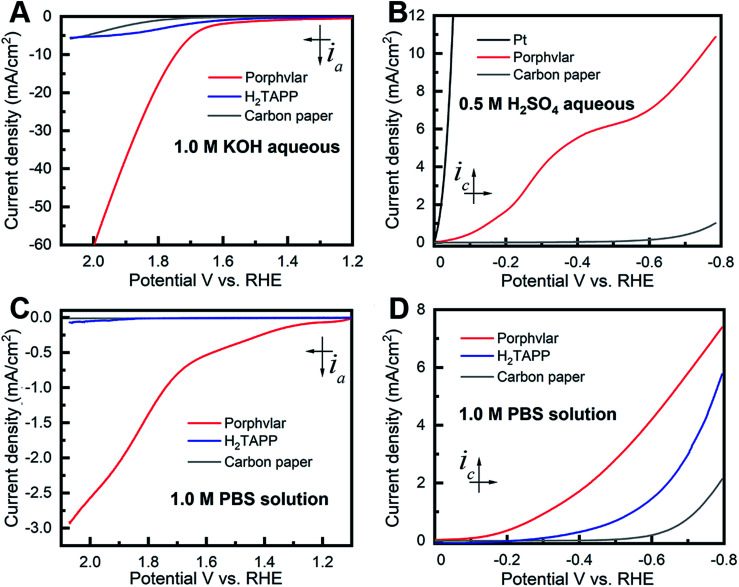
Polarization curves of molecular porphyrin (H_2_TAPP) (blue), Porphvlar (red) and blank carbon paper electrode under different conditions. (A) Oxidation in 1.0 M KOH aqueous solution; (B) reduction in 0.5 M H_2_SO_4_ aqueous solution, (C) oxidation in 1.0 M PBS buffer solution; (D) reduction in 1.0 M PBS buffer solution, scan rate: 5 mV s^−1^.

The current response of Porphvlar reaches a current density of 10 mA cm^−2^ at 1.75 V (*η*_10_ = 520 mV) in basic electrolyte, while the molecular porphyrin plateaus at 5 mA cm^−2^ (Fig. 3A). The electrocatalytic OER performance for Porphvlar was also investigated in neutral PBS. As depicted in Fig. 3C, the current density of Porphvlar increases slowly at 1.31 V *vs.* RHE until 1.63 V *vs.* RHE. Porphvlar requires an overpotential of 520 mV to achieve a current density of 1 mA cm^−2^, however, the molecular H_2_TAPP shows negligible current increase under the same conditions. An electrode was prepared with commercial PPTA thread and was used as a working electrode, the low current density show PPTA is inactive for both HER and OER (Fig. S7†). Therefore, the current density corresponds to catalytic electrochemical activity when porphyrin units are incorporated into PPTA networks. The generated gas obtained from the 60 h Porphvlar bulk electrolysis was collected in a gas-tight H-type cell and was confirmed to be O_2_ by gas chromatography with 92.7% faradaic efficiency in 1.0 M KOH and 84.5% faradaic efficiency in 1.0 M PBS solution.

Fig. 3B shows the HER electrocatalytic activity of Porphvlar in a 0.5 M H_2_SO_4_ aqueous solution. The Pt electrode is shown as a benchmark for HER. Porphvlar has an onset potential of 36 mV *vs.* RHE and reaches a current density of 1 mA cm^−2^ at 144 mV. H_2_TAPP does not perform HER in 0.5 M H_2_SO_4_ aqueous solution due to the protonation reaction in the nitrogen core. A pre-wave was observed at around −0.4 V *vs.* RHE, which is a phenomenon of adsorption of protons that accumulate favorably on the modified electrode surface. The competition between the adsorption stage and the cathodic reduction is significant but consecutively since the pre-wave disappears when precludes the mass transfer factor (Fig. S13B†). The collected reduction product was confirmed to be hydrogen gas by gas chromatography with a faradaic efficiency of 98% in acidic electrolyte and 99.55% in PBS buffer.

Additionally, the porphyrin unit was metallated with zinc using standard synthetic procedures (see ESI†). Fig. S23† shows Zn-Porphvlar exhibits a considerably lower current response towards HER and OER with respect to the corresponding metal-free materials. This suggests that the catalytic center is located at the four-nitrogen core in the free-base porphyrin units. Thus, incorporating a redox inactive metal ion such as Zn can effectively shut down the activity of Porphvlar towards catalyzing water splitting electrochemically.

**Fig. 4:**
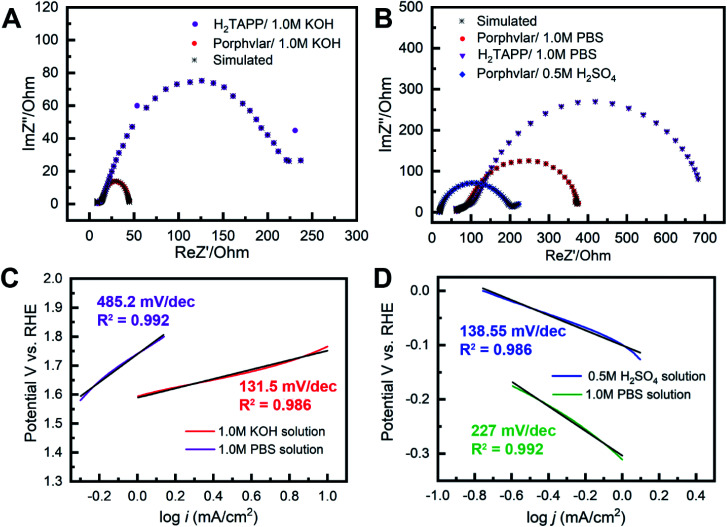
Electrochemical impedance spectroscopy of Porphvlar (red) and H_2_TAPP (purple) operated at 250 mV overpotential: (A) in 1.0 M KOH aqueous solution (B) in 1.0 M PBS buffer solution and 0.5 M H_2_SO_4_ aqueous solution (blue), respectively; Tafel plots of Porphvlar constructed by polarization curves: (C) in 1.0 M KOH aqueous solution (red) and 1.0 M PBS buffer (purple); (D) 1.0 M PBS buffer solution (green) and 0.5 M H_2_SO_4_ aqueous solution (blue).

The kinetic properties of Porphvlar and H_2_TAPP under oxidation and reduction conditions within different electrolytes were characterized by electrochemical impedance spectroscopy (EIS) in order to study the interface charge transport process. The Nyquist plots of Porphvlar and H_2_TAPP, obtained by measuring the parametric response of the imaginary part *vs.* the real part of the impedance, are shown in Fig. 4A and B. The diameter of the semicircle relates to the electron transfer process from the electrode surface into the electrolyte solution since it arises from the parallel combination of faradaic charge transfer resistance and non-faradaic double-layer capacitance. Therefore, the smaller the semicircle the less the charge transfer resistance.^44,45^ It can be seen that the semicircle in the high-frequency range of the Nyquist plot of Porphvlar has a smaller diameter than that of the molecular porphyrin unit under both oxidation and reduction conditions. This suggests that the Porphvlar network favors charge transfer during water splitting. Furthermore, the increased electron transfer from the modified electrode to the electrolyte enhances the catalytic activity towards both HER and OER.

EIS analyses were performed to investigate the Porphvlar cathode/anode interphase during the water splitting reaction and modeled with the appropriate equivalent circuit (Fig. S8 and S9†). Experimental results fit to the Randles circuit and fitted parameters are shown in Table S1.† Constant phase elements (CPEs) are used for the non-ideal capacitance from the non-homogeneous electrode surface with the ideality factor *n*. *R*_1_ is the series resistance for the electrolyte and the electrode materials. The simulation shows that for HER the electrochemical processes are different when the reaction happens in acidic or neutral conditions (Fig. S8C and F†). For the HER in sulfuric acid, the parallel branch (CPE_1_/(*R*_2_ + *W*)) indicates that the interphase between the electrode and the electrolyte is where electron transfer occurs, and *R*_2_ represents the charge transfer resistance (*R*_ct_). In PBS buffer, HER requires two steps: the first branch describes the contact between the electrode and Porphvlar; the second branch (CPE_3_/*R*_3_) is responsible for the double-layer impedance and the charge transfer resistance at the interphase of the solvent and Porphvlar. For OER, Porphvlar has a similar two-step catalytic process (Fig. S9C and F†) in both basic conditions and PBS buffer. An extra diffusion impedance element (Warburg diffusion) is included in the simulation in a neutral solution since the reaction is no longer controlled by kinetics due to the lack of readily available hydroxide ions. In these conditions, the reaction rate depends on the diffusion of buffering ions. As result, buffered ions penetrate the diffusion layer creating a finite thickness Warburg element.

To evaluate the kinetics during the electrolysis process, Tafel plots were constructed from polarization curves (Fig. 4C and D). The linear portions of the Tafel plots were fit to the Tafel equation: *η* = *a* + *b* log(*j*), where *η* is the overpotential, *j* is the current density, and *b* is the Tafel slope. The calculated Tafel slope for Porphvlar (131.5 mV dec^−1^) in a 1.0 M KOH solution is comparable to that of the benchmark OER electrocatalyst IrO_2_ (83 mV dec^−1^),^46^ suggesting fast reaction kinetics and high OER activity. In 0.5 M H_2_SO_4_ solution, Porphvlar has an HER Tafel slope of 138.55 mV dec^−1^, which also indicates efficient kinetics. The general HER mechanism in metal surfaces has been extensively studied, and three mechanisms have been proposed for the different Tafel slopes: Volmer (120 mV dec^−1^), Heyrovsky (40 mV dec^−1^) and Tafel (30 mV dec^−1^).^47^ The Porphvlar HER Tafel slope (in neutral and acidic conditions) suggests a Volmer–Heyrovsky mechanism *via* water reduction H_2_O + e^−^ = H_ads_ + OH^−^ and H_2_O + e^−^ + H_ads_ = H_2_ + OH^−^.^48^ The Tafel slopes are larger for HER and OER when using PBS as an electrolyte, these slow kinetics might be due to the low ion concentration and pH in the reaction.

**Fig. 5:**
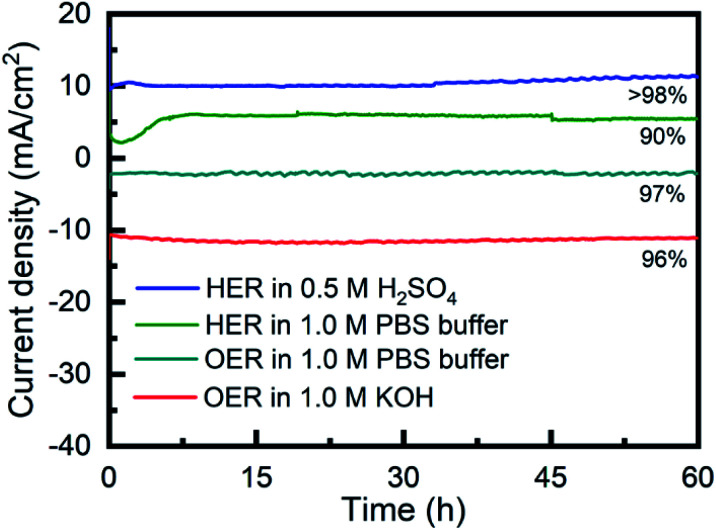
Time dependence of the current density for as-synthesized Porphvlar at static potential; blue: HER in 0.5 M H_2_SO_4_; green: in 1.0 M PBS buffer; teal: OER in 1.0 M PBS buffer; red: OER in 1.0 M KOH.

The long-term stability of Porphvlar in different electrolytes under both oxidation and reduction conditions, was studied with bulk electrolysis for 60 hours. Fig. 5 shows that Porphvlar exhibits constant and stable anodic and cathodic currents during OER and HER electrocatalysis, respectively, during this timeframe. Energy-dispersive X-ray spectroscopy mapping shows no metal contamination or deposition on the Porphvlar network after the electrolysis in basic and acidic conditions mentioned (Fig. S2–S4†). The FT-IR and UV-vis spectra of the Porphvlar material/electrode and the corresponding electrolyte before and after the bulk electrolysis show unchanged characteristic peaks (Fig. S10–S12†). The durability of Porphvlar can be attributed to the stability of the conjugated porphyrin networks and the hydrogen bonding within its layers that result in a unique catalytic material for HER and OER.

## Conclusions

In summary, an efficient and stable metal-free electrocatalyst for HER and OER has been developed. This bifunctional material was constructed through the condensation of (4-aminophenyl) porphyrin (H_2_TAPP) into PPTA networks. The HER and OER catalytic performances are higher than those of other metal-free materials and comparable or better than several traditional metallic electrocatalysts. The catalytic ability of Porphvlar which occurs at the molecular porphyrin nodes is further enhanced by the 2D network arrangement of the hydrogen-bonded PPTA networks. This material provides a novel approach to the design of metal-free electrocatalysts and takes advantage of the structural interactions within a 2D network to yield high electrochemical performance, conductivity, and durability.

## Experimental

### Materials and methods

4-Nitrobenzaldehyde (99%), tin(ii) chloride dihydride (SnCl_2_·2H_2_O, 98+%) were purchased from Acros Organics, terephthaloyl chloride (99%) and Nafion solution (5% w/w in H_2_O and 1-propanol) were obtained in Alfa Aesar, pyrrole (98%), pyridine (99%), sulfuric acid (H_2_SO_4_), potassium hydroxide (KOH) and 1.0 M PBS buffer (pH = 7) were purchased from Fisher Scientific and used without further purification. All the glassware and cells were decontaminated by soaking in aqua regia solution (conc. HCl/conc. nitric acid = 3 : 1). The glass apparatus was washed with deionized water and oven dried. Carbon paper substrate was purchased from Fuel Cell Store (AvCarb P75T −40 × 40 cm), which is carbon particle-filled, polyacrylonitrile (PAN) based carbon fiber paper with hydrophobic Teflon wet-proof (13% wt treatment) coating nominal thickness of 0.255 mm (@ 1 psi/0.7 N cm^−2^).

### Electrochemical methods

All electrochemical measurements were performed in a three-compartment electrochemical glass cell using a CHI760D potentiostat. A graphite rod (2 mm, radius; 40 mm, length; 515 mm^2^ surface area) was used as the counter electrode, and a saturated calomel electrode (SCE) was employed as the reference electrode. Aqueous 1.0 M KOH, 0.5 M H_2_SO_4_ and 1.0 M PBS buffer were used as electrolytes and purged with nitrogen gas to remove the dissolved oxygen before each measurement. To minimize the double layer charging, a low scan rate of 5 mV s^−1^ was used to perform linear sweep voltammetry (LSV). Electrochemical impedance spectroscopy (EIS) was obtained at an overpotential, *η*, of 250 mV from 100 kHz to 0.1 Hz with an AC voltage of 5 mV. Bulk electrolysis and chronoamperometric measurements were tested at the voltage with current density at around 10 mA, 5 mA, and 1 mA for 60 hours in ambient atmosphere. All potentials referenced to saturated calomel electrode (SCE) were calibrated with respect to reversible hydrogen electrode (RHE) using the equation: *E* = *E*_0_ + 0.245 + 0.059 × pH. All the experimental potential data were calibrated at a pH of 14 for basic conditions and pH of 7 for neutral conditions and were performed at ambient temperature. All the current densities obtained were normalized by dividing the obtained current response by the geometric area of the working electrode (0.25 cm^2^).

### Instrumentation

Porphvlar samples were characterized by scanning electron microscopy (SEM), energy dispersive X-ray spectroscopy (EDX), and powder X-ray diffraction (pXRD). SEM and EDX studies were performed on an SEM Hitachi S-4800 instrument. Powder X-ray diffraction pXRD patterns were obtained on a Panalytical Empyrean X-ray diffractometer. The structural characterizations were further elucidated by infrared spectroscopy (IR) through an Agilent Cary 630 FT-IR spectrometer and UV-vis spectra data were obtained with an ALS SEC2020 spectrometer system. Elemental analyses measurements were performed with a PerkinElmer 4300 DV inductively-coupled plasma optical-emission spectrometer (ICP-OES). Porphvlar samples were digested using aqua regia at 115 °C for 45 min before measurement.

### Preparation of catalyst modified carbon paper electrodes

A catalyst ink was prepared by mixing catalyst powder (5.0 mg), carbon black (0.25 mg), 2-isopropanol (1.00 mL) and a Nafion solution (4.08 μL). The mixture was ultrasonicated for 30 min to generate a homogenous dispersion. Carbon paper was cut into 0.5 cm × 2 cm strips and dried at 30 °C for 12 hours in air before use. 10 μL of the as-prepared catalyst ink was drop-casted on the carbon paper and was allowed to dry in ambient atmosphere for 10 minutes before each measurement. Electrodes using the molecular H_2_TAPP catalyst were prepared using an identical method.

### Sample preparation for SEM/EDX analysis

The Porphvlar sample was carefully mounted on aluminum stubs (12 mm) using conductive carbon tapes (Electron Microscopy Science, 125 μm thickness) without coating.

### Gas determination

Bulk electrolysis was performed in an H-type divided cell (120 mL) with a frit separating the two half reactions. A graphite rod electrode (2 mm, radius; 40 mm, length; 515 mm^2^ surface area) was as the counter electrode. The electrochemistry cell was purged with high purity argon gas before electrolysis. Quantitative gaseous products were measured online by a gas chromatography instrument (SHIMADZU GC-8A) equipped with a Zebron ZB-1 column (30 m (length), 0.53 mm (I.D), 5 μm (film thickness)) and high-purity argon (99.999%) was used as the carrier gas. Faradaic efficiency (FE) is calculated as follows: FE = *αnFIt* × 100% where *α* = 2 for the number of electrons transferred to generate hydrogen and *α* = 4 for the number of electrons transferred to generate oxygen; *F* is the faradaic constant (96 485 C mol^−1^); *n* is the amount of gas generated in mols (7.69 μmol for OER in 1.0 M KOH; 2.311 μmol for OER in 1.0 M PBS buffer; 1.8 μmol for HER in 0.5 M H_2_SO_4_; 1.37 μmol for HER in 1.0 M PBS buffer); *I* (A) is the current and *t* (s) is the time duration for the bulk electrolysis.

## Data availability

The datasets supporting this article have been uploaded as part of the ESI.†

## Author contributions

D. Villagran and Y. Ge conceived and designed the experiments. Z. Lyu and Y. Ge synthesized the materials, performed the FT-IR and UV-vis characterization and electrochemical experiments. M. Marcos-Hernandez performed the p-XRD, and SEM-EDX mapping measurements. All authors analyzed the data, discussed the results, and reviewed the manuscript.

## Conflicts of interest

There are no conflicts to declare.

## Supplementary Material

SC-013-D2SC01250B-s001
