# Supplementary material for: Free-base porphyrin polymer for bifunctional electrochemical water splitting
Source: Chem Sci. 2022 Jul 4;13(29):8597–604. doi: 10.1039/d2sc01250b (PMC9337729; doi:10.1039/d2sc01250b)
Supplement: SC-013-D2SC01250B-s001 [file SC-013-D2SC01250B-s001.pdf]

Electronic Supplementary Material (ESI) for Chemical Science.
This journal is © The Royal Society of Chemistry 2022

Supplementary Information for:

Free-base Porphyrin Polymer for Bifunctional Electrochemical Water Splitting

Yulu Ge, Zhenhua Lyu, Mariana Marcos-Hernández and Dino Villagrán*

* Dino Villagrán
Email: dino@utep.edu

This PDF file includes:

Supplementary text
Figures S1 to S23
Scheme S1 to S2
Table S1

SI References

Table of Contents

Fig. S1. Energy-dispersive X-ray spectroscopy (EDX) mapping of Porphvlar sample.......4

Fig. S2. Energy-dispersive X-ray spectroscopy (EDX) mapping of Porphvlar modified electrode...5

Fig. S3. Energy-dispersive X-ray spectroscopy (EDX) mapping of Porphvlar modified electrode after the long-term electrolysis for HER. 6

Fig. S4. Energy-dispersive X-ray spectroscopy (EDX) mapping of Porphvlar modified electrode after the long-term electrolysis for OER 7

Fig. S5. Energy-dispersive X-ray spectroscopy (EDX) mapping of Zn-Porphvlar.............8

Fig. S6. High resolution XPS spectra of Porphvlar...9

Fig. S7. Polarization curves of commercial PPTA thread and Porphvlar electrode........10

Fig. S8. Electrochemical impedance spectroscopy (EIS) analysis of hydrogen evolution reaction catalyzed by Porphvlar 11

Fig. S9. Electrochemical impedance spectroscopy (EIS) analysis of oxygen evolution reaction catalyzed by Porphvlar 12

Table S1. Parameters for Equivalent Circuits for the water splitting reaction reported in this work. 13

Fig. S10. Overlaid UV-vis spectra Porphvlar before and after bulk electrolysis 14

Fig. S11. Overlaid Infrared spectra of Porphvlar combined with carbon black before and after bulk electrolysis 15

Fig. S12. Overlaid UV-vis spectra of electrolyte before and after long-term electrolysis using Porphvlar working electrode 16

Fig. S13. Overlaid cyclic voltammograms of Porphvlar using a rotating disk electrode17

Fig. S14. Overlaid linear sweep voltammograms of Porphvlar sample with and without Nafion® in the dropcast sample ink 18

Fig. S15. Overlaid linear sweep voltammogram of Porphvlar before and after 500 cycles. 19

Fig. S16. Overlaid linear sweep voltammogram of the rinsed Porphvlar electrode and the Porphvlar electrode after 500 cycles 20

Electrochemical Active Surface Area (ECSA). 21

Fig. S17. Cyclic voltammograms of as-prepared Porphvlar at different scan rate in 0.1 V OCP potential window...22

Fig. S18. Linear sweep voltammogram of Porphvlar with current density normalized with ECSA...24

Fig. S19. Time dependence of the current density for molecular porphyrin (H_2TAPP) at static potential in 1.0 M KOH for 60 hours on carbon paper electrode. 25

Scheme S1. Synthesis procedure of H_2TAPP. 26

Fig. S20. ^{1}H NMR spectrum of 5,10,15,20-tetrakis(4-aminophenyl) porphyrin (H_2TAPP) in DMSO-D6. 27

Scheme S2. Synthesis procedure of ZnTAPP. 28

Fig. S21. ^{1}H NMR spectrum of Zn(II) 5,10,15,20-tetrakis(4-aminophenyl) porphyrin (ZnTAPP) in DMSO-D6. 29

Synthesis of Porphvlar 30

Synthesis of Zn-Porphvlar 30

Fig. S22. Spectroscopy characterization of Zn-Porphvlar.. 31

Fig. S23. Comparison linear sweep voltammetry of Zn-Porphvlar with the metal-free Porphvlar in different electrolytes. 32

SI References 33

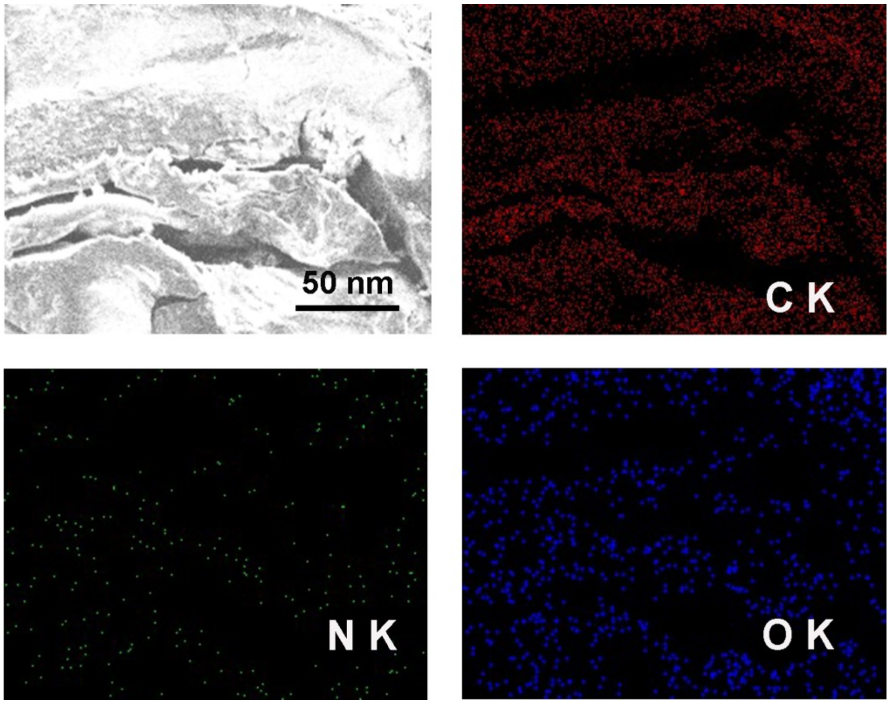

Fig. S1. Energy-dispersive X-ray spectroscopy (EDX) mapping of Porphvlar sample.

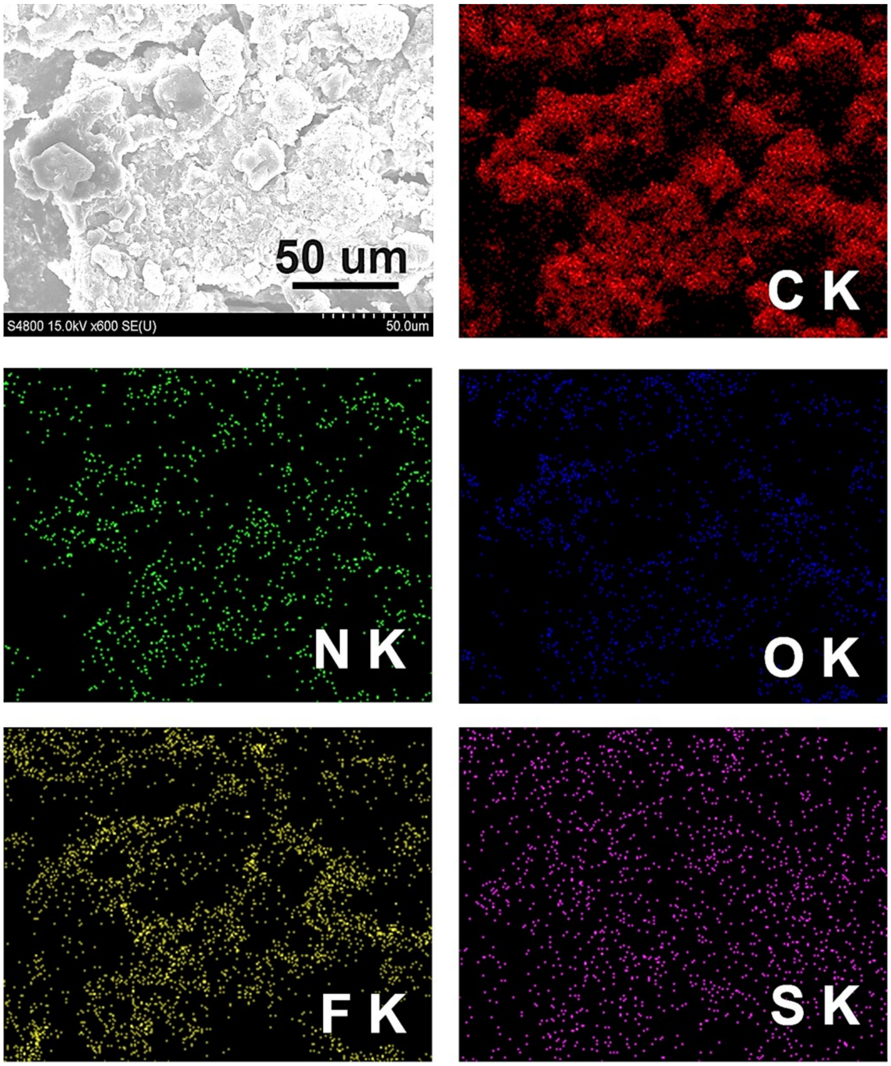

Fig. S2. Energy-dispersive X-ray spectroscopy (EDX) mapping of Porphvlar modified electrode. The elemental mapping shows no metal content in Porphvlar working electrode. The F and S element appear in the mapping also, which serve as evidence for the Nafion® ($C_7HF_{13}O_5S \bullet C_2F_4$) as the conductive binder during the electrode preparation. Trace of Cl element was found in the mapping.

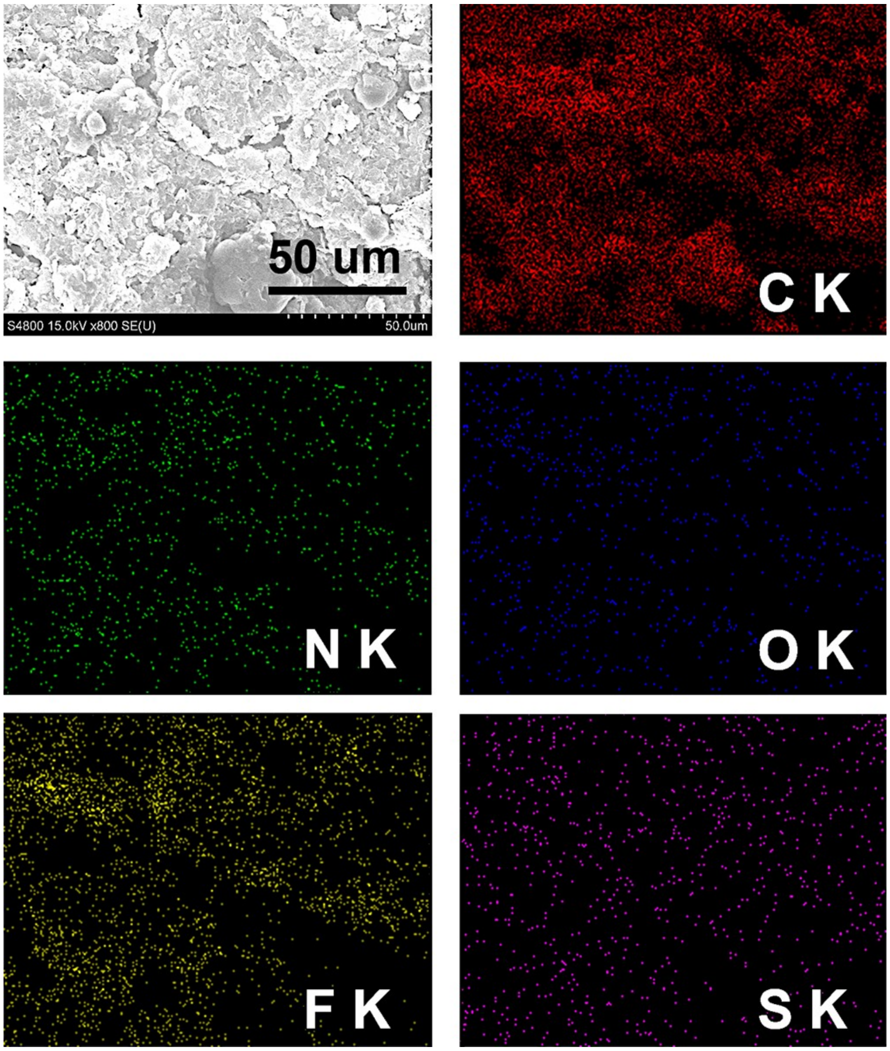

Fig. S3. Energy-dispersive X-ray spectroscopy (EDX) mapping of Porphvlar modified electrode after the long-term electrolysis for hydrogen evolution reaction in 0.5 M H_2SO_4 aqueous solution for 60 hours. The elemental mapping shows no metal contamination or deposition on the Porphvlar working electrode during or after the electrolysis. The F and S element appear in the mapping also, which serve as evidence for the Nafion® ($C_7HF_{13}O_5S$ • C_2F_4) as the conductive binder during the electrode preparation. Trace of Cl element was found in the mapping.

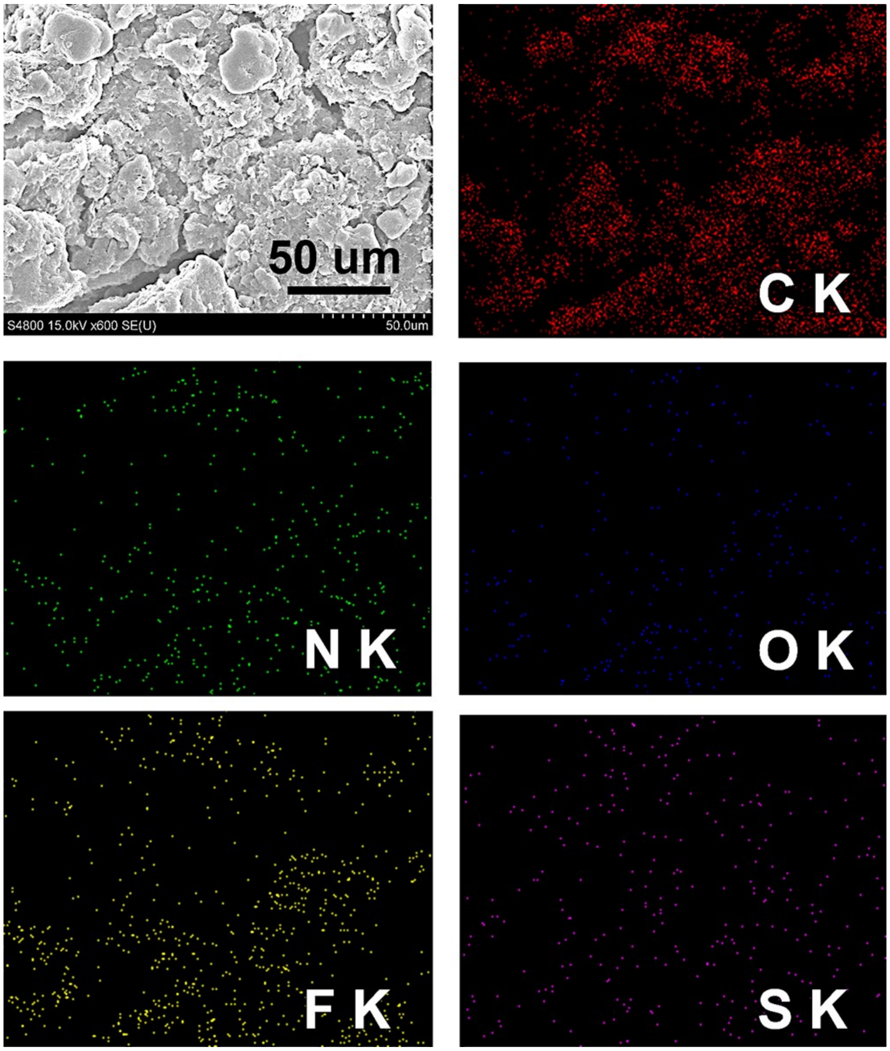

Fig. S4. Energy-dispersive X-ray spectroscopy (EDX) mapping of Porphvlar modified electrode after the long-term electrolysis for oxygen evolution reaction in 1.0 M KOH aqueous solution for 60 hours. Same as the description in Fig. 2S, no metal was found during or after the electrolysis due to the possible contamination or deposition. F and S elements belong to the Nafion® binder and trace amount of Cl was also found in the mapping.

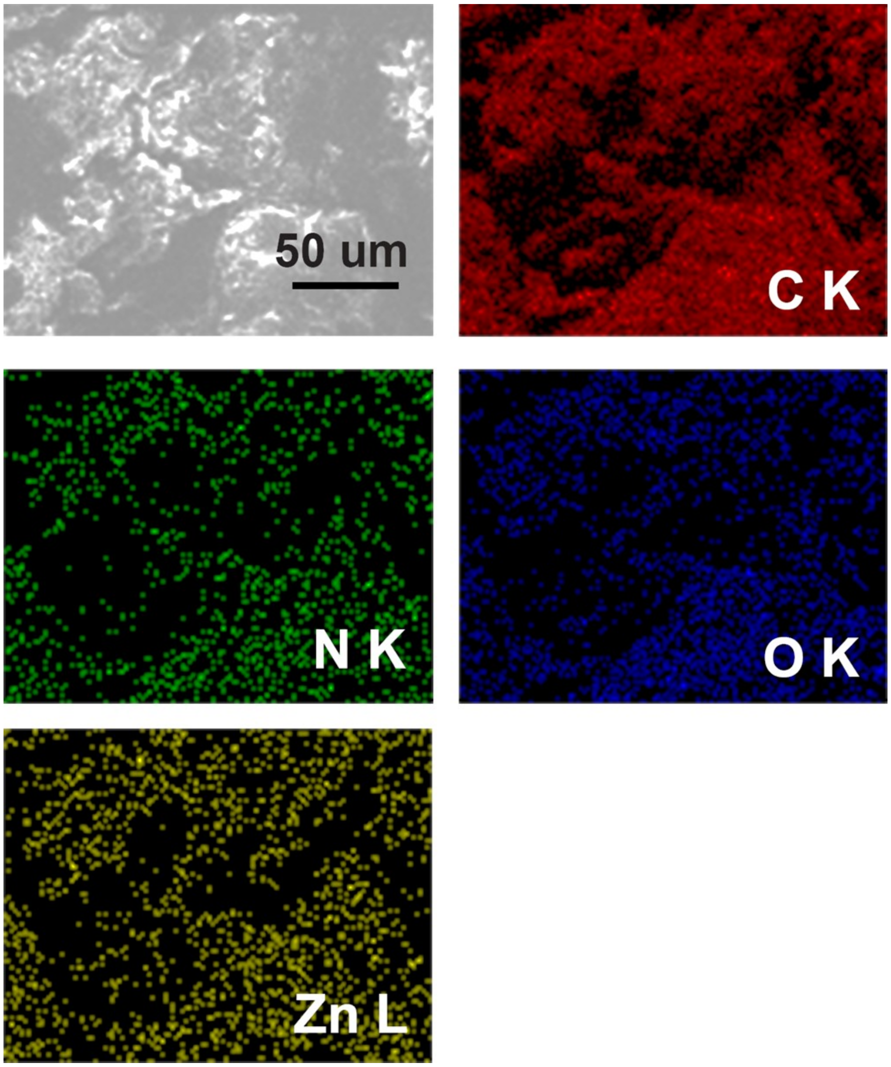

Fig. S5. Energy-dispersive X-ray spectroscopy (EDX) mapping of Zn-Porphvlar, trace amount of Cl was also found in this mapping.

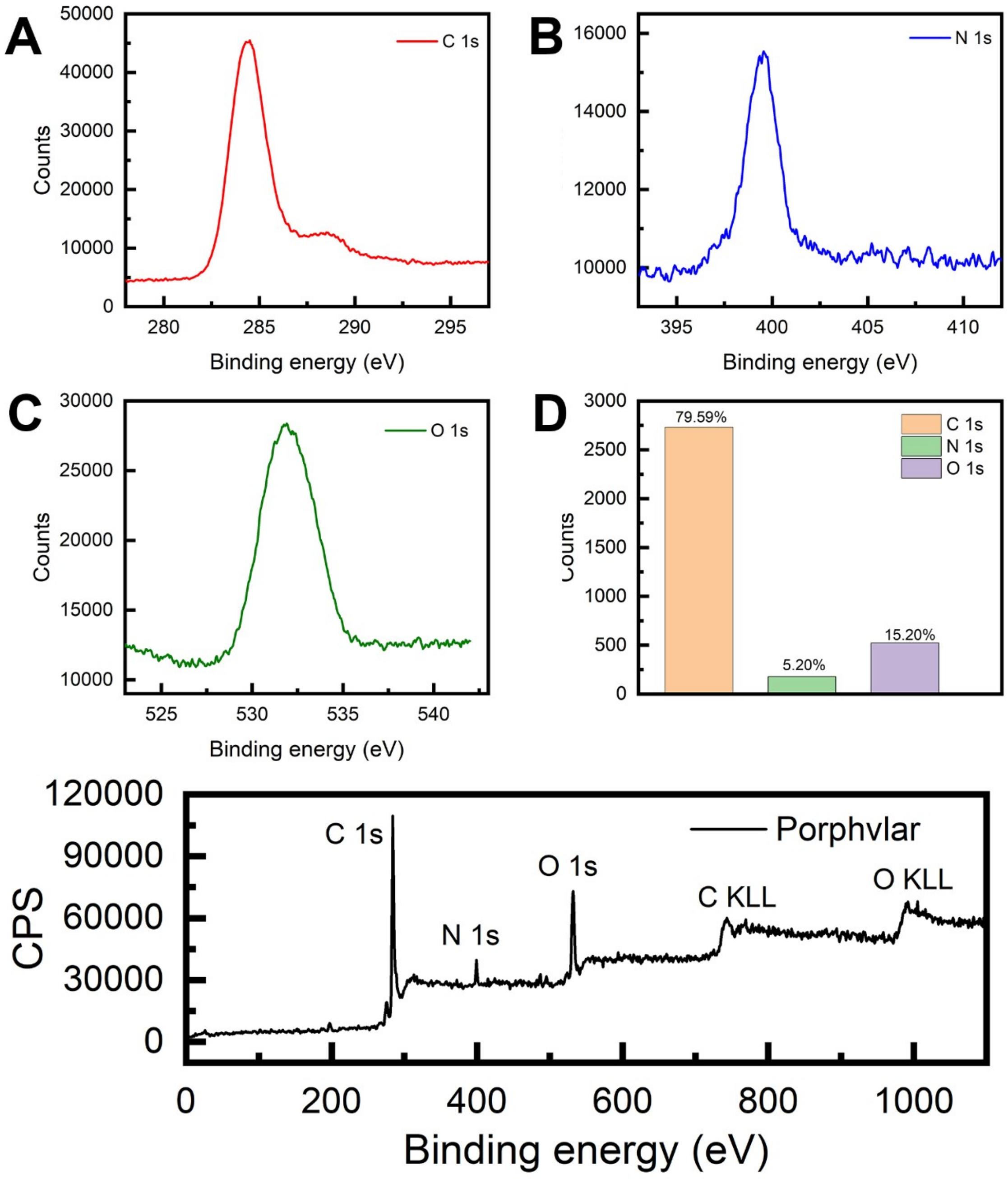

Fig. S6. High resolution XPS spectra of Porphvlar sample (A-C); (D). Atomic ratio of C, N, and O of the Porphvlar sample; (E) Full XPS survey.

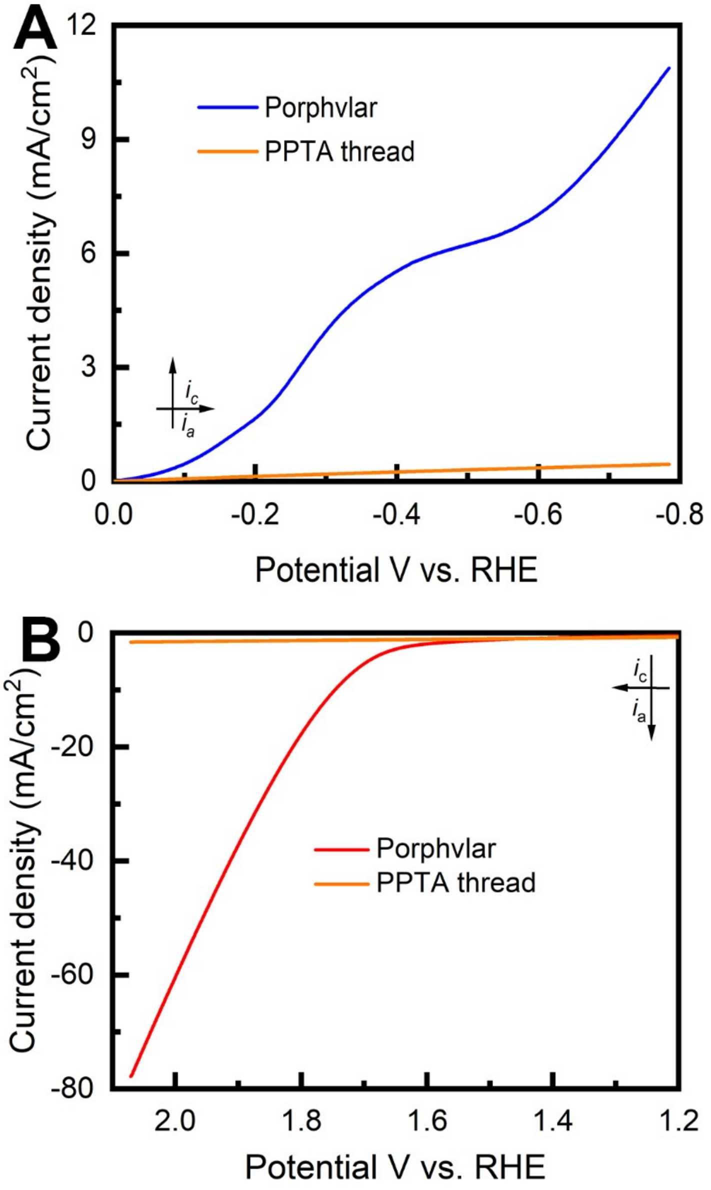

Fig. S7. Polarization curves of commercial PPTA thread (orange) and Porphvlar (red) as working electrodes under the same conditions on carbon paper electrode: (a). Oxidation in 1.0 M KOH, scan rate: 5 mV/s, (b). Reduction 0.5 M H_2SO_4 solution, scan rate: 5 mV/s. No catalytic current is shown in both HER and OER for the PPTA thread as working electrode compared with Porphvlar in the corresponding electrolytes.

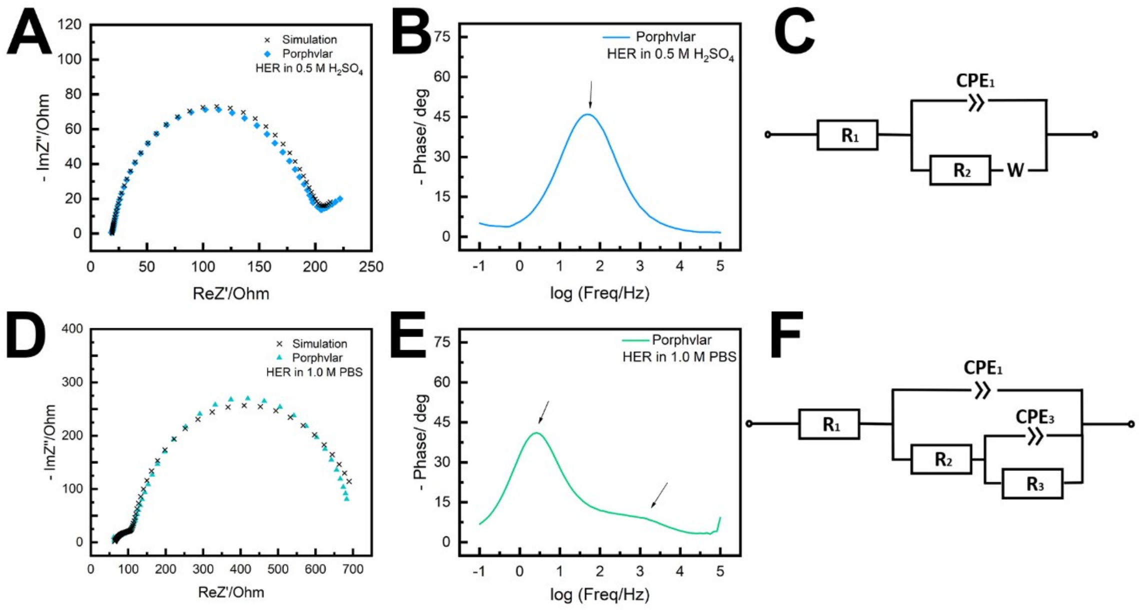

Fig. S8. Electrochemical impedance spectroscopy (EIS) of hydrogen evolution reaction catalyzed by Porphvlar on carbon paper electrode: (A) Nyquist plot of HER in 0.5 M H_2SO_4 aqueous solution with simulation fit. (B) Bode plot of HER in 0.5 M H_2SO_4 aqueous solution. (C) The electrical equivalent circuit (EEC) used to simulate the impedance spectra fit in 0.5 M H_2SO_4 aqueous solution. (D) Nyquist plot of HER in 1.0 M PBS solution with simulation fit. (E) Bode plot of HER in 1.0 M PBS solution. (F) The electrical equivalent circuit (EEC) used to simulate the impedance spectra fit in 1.0 M PBS solution.

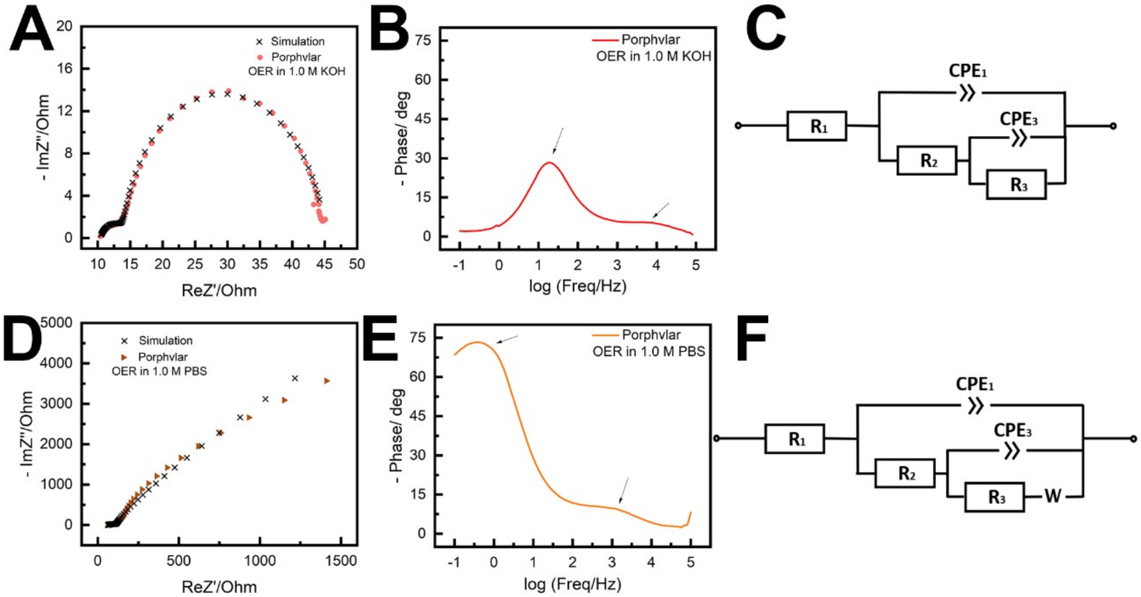

Fig. S9. Electrochemical impedance spectroscopy (EIS) of oxygen evolution reaction catalyzed by Porphvlar on carbon paper electrode: (A) Nyquist plot of OER in 1.0 M KOH aqueous solution with simulation fit. (B) Bode plot of OER in 1.0 M KOH aqueous solution. (C) The electrical equivalent circuit (EEC) used to simulate the impedance spectra fit in 1.0 M KOH aqueous solution. (D) Nyquist plot of OER in 1.0 M PBS solution with simulation fit. (E) Bode plot of OER in 1.0 M PBS solution. (F) The electrical equivalent circuit (EEC) used to simulate the impedance spectra fit in 1.0 M PBS solution.

Table S1. Parameters for Equivalent Circuits for the water splitting reaction reported in this work.

	**Porphvlar**				**H_2TAPP**		
	HER		OER		HER	OER	
**Electrolyte**	0.5 M H_2SO_4	1.0 M PBS	1.0 M KOH	1.0 M PBS	1.0 M PBS	1.0 M KOH	1.0 M PBS
**R1 (Ohm)**	18.84	62.60	10.34	60.73	61.99	8.919	63.15
**CPE1 (F.s^(a-1))**	N/A	0.1611e-3	0.2996e-3	0.1549e-3	58.29e-3	0.1591e-3	58.33e-6
**n1**	N/A	0.6385	0.6902	0.6349	0.6688	0.6888	0.6845
**CPE2/3 (F.s^(a-1))**	0.1267e-3	0.203e-3	0.506e-3	0.1549e-3	0.2321	0.67e-3	0.1193e-3
**n2/3**	0.8612	0.9557	0.9495	0.9365	0.0965	0.8398	0.8583
**R2 (Ohm)**	180.5	67.56	4.415	69.06	1098	6.098	43.08
**R3 (Ohm)**	N/A	622.8	30.67	31.66	303.0	206.6	477.5
**s2 (Ohm.s^1/2)**	11.99	N/A	N/A	N/A	22.64	N/A	N/A
χ^2	0.03369	0.03336	9.155e-3	0.07571	0.0306	0.2432	0.08639
$\chi/\sqrt{N}$	0.0215	0.0225	0.0124	0.0322	0.0214	0.0598	0.0243

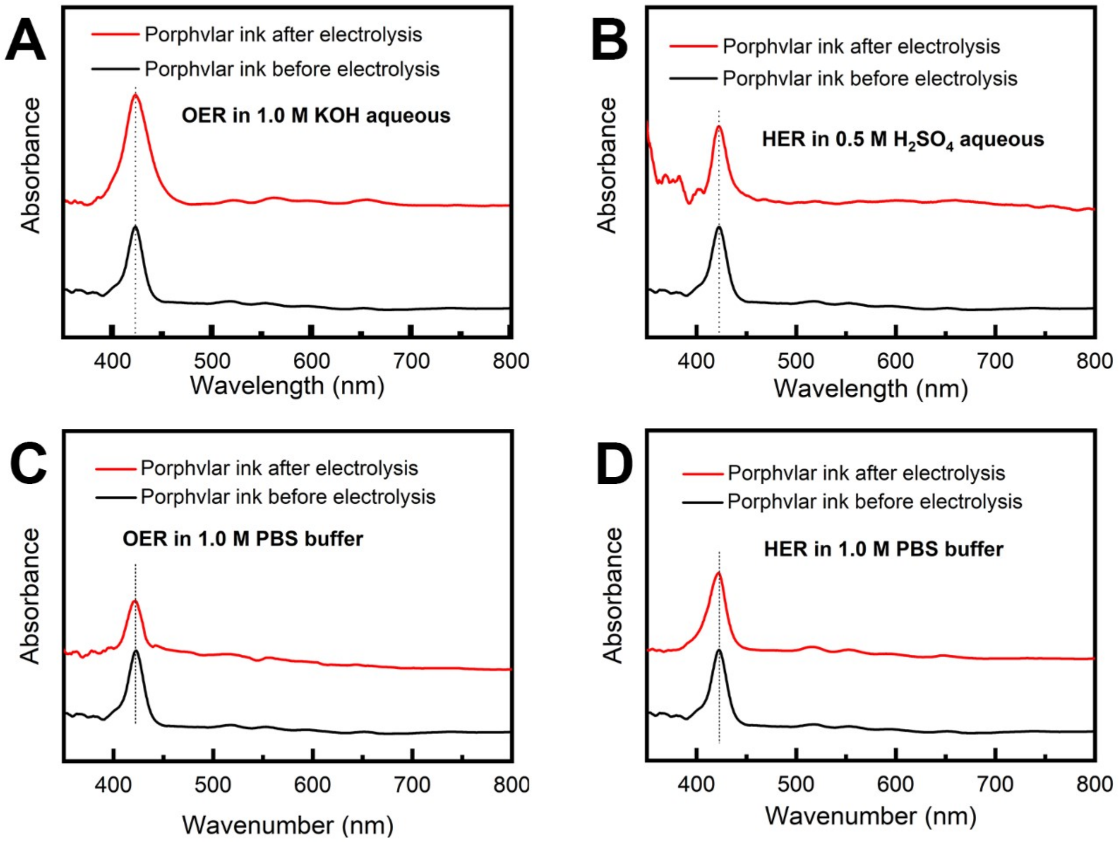

Fig. S10. Overlaid UV-vis spectra Porphvlar combined with carbon black in EtOH suspension before (black) and after (red) bulk electrolysis after 60 hours in: (A). OER 1.0 M KOH aqueous solution; (B). HER in 0.5 M H_2SO_4 aqueous solution; (C). OER in 1.0 M PBS buffer; (D). HER in 1.0 M PBS buffer. The UV-vis spectra do not change after the long-term bulk electrolysis, indicating the Porphvlar sample maintained its chemical structure during the electrolysis.

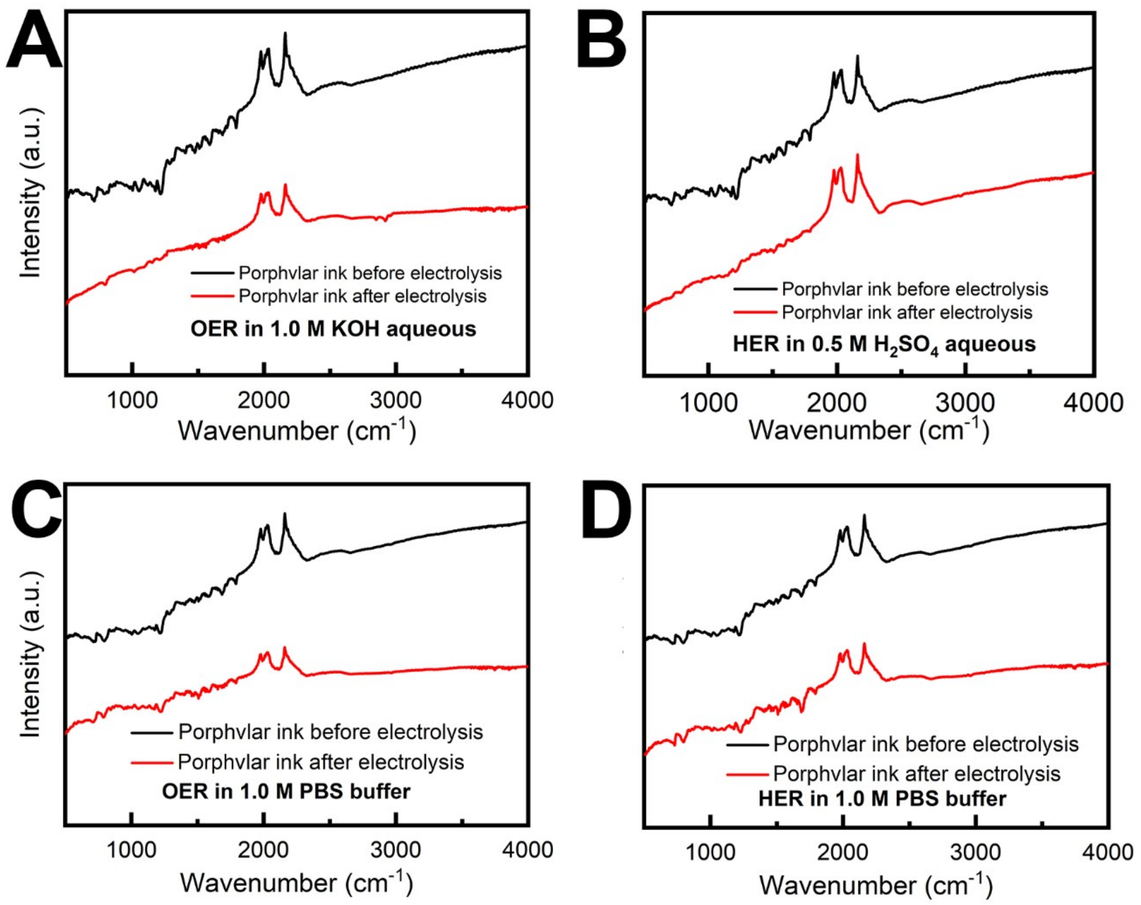

Fig. S11. Overlaid Infrared spectra of Porphvlar combined with carbon black before (black) and after (red) bulk electrolysis after 60 hours in: (A). OER 1.0 M KOH aqueous solution; (B). HER in 0.5 M H_2SO_4 aqueous solution; (C). OER in 1.0 M PBS buffer; (D). HER in 1.0 M PBS buffer. The Infrared spectra do not change before and after the bulk electrolysis, indicating the unchanged chemical component of the Porphvlar sample after long-term electrolysis.

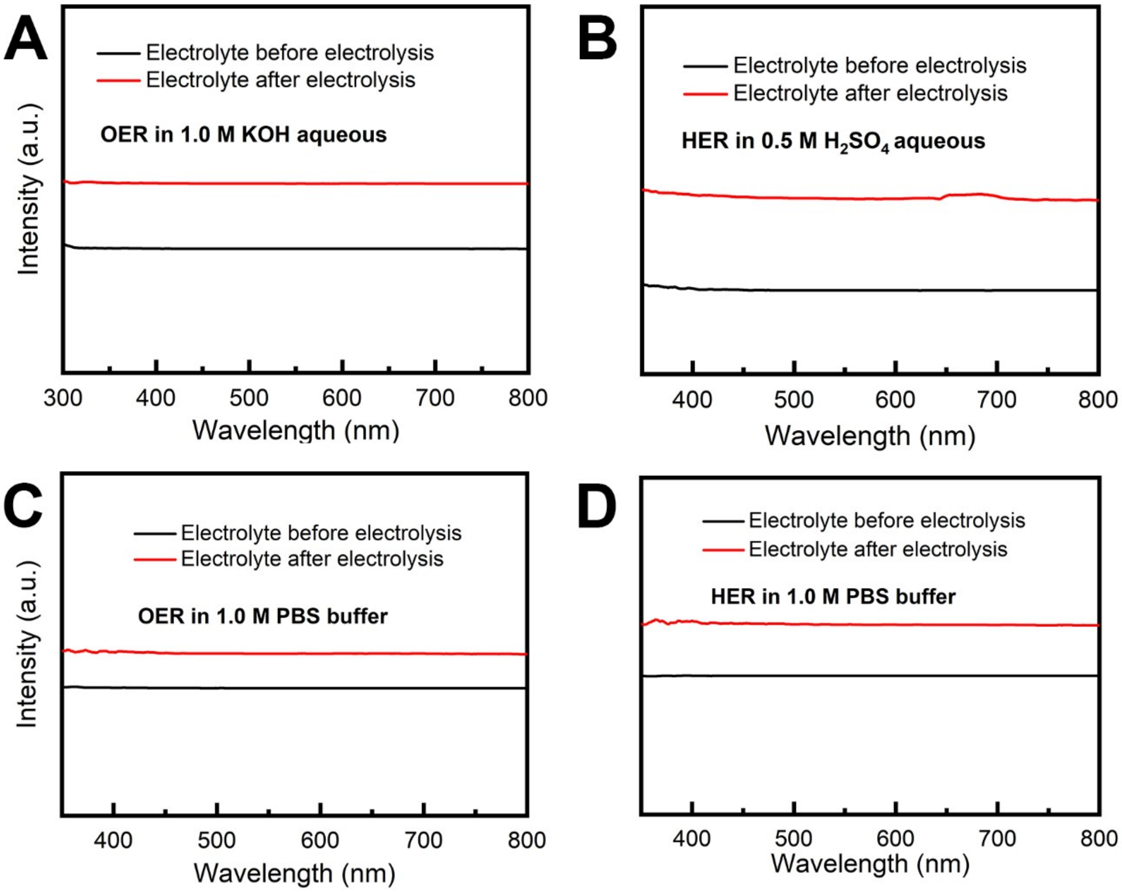

Fig. S12. Overlaid UV-vis spectra of electrolyte before and after 60 h long-term electrolysis using Porphvlar modified carbon paper working electrode: (A) OER in 1.0 M KOH aqueous solution; (B) HER in 0.5 M H_2SO_4 aqueous solution; (C) OER in 1.0 M PBS buffer; (D) HER in 1.0 M PBS buffer. No peaks appear in the visible region after the electrolysis, indicating no leaching of porphyrin units or decomposition during the electrolysis.

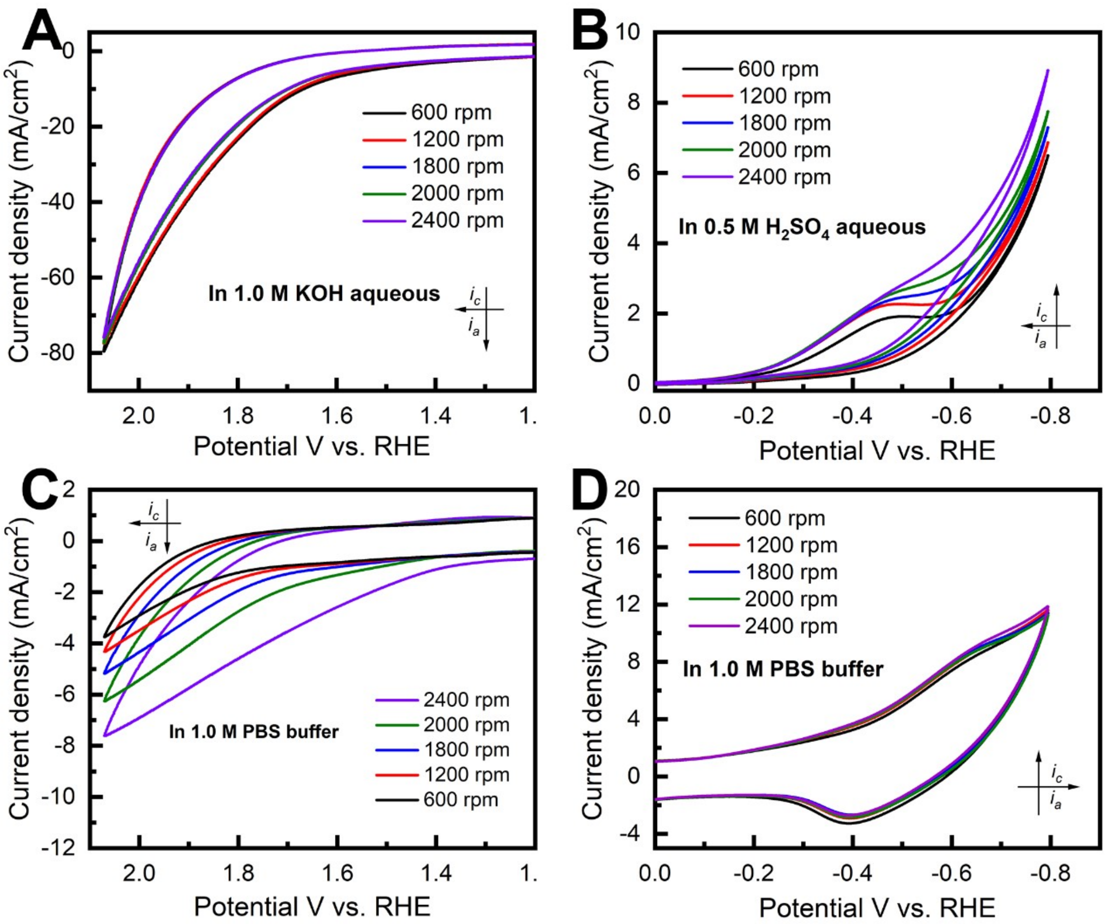

Fig. S13. Overlaid cyclic voltammograms of Porphvlar using a rotating disk electrode (RDE): (a) OER 1.0 M KOH aqueous solution; (b) HER in 0.5 M H_2SO_4 aqueous solution; (c) OER in 1.0 M PBS buffer; (d) HER in 1.0 M PBS buffer. Rotating disk electrode measurements: a glassy carbon disc of 5 mm in diameter (Pine Instrument) was employed as working electrode. All electrochemical experiments were performed using a potential range from 1.2 to 2.1 V vs. RHE at a scan rate of 5 mV/s for OER and 0 to −0.8 V vs. RHE at a scan rate of 5 mV/s for HER.

Rotating disk electrode is utilized for testing the catalytic performance of the Porphvlar sample in order to minimize the diffusion layer thickness in a slow scan rate.

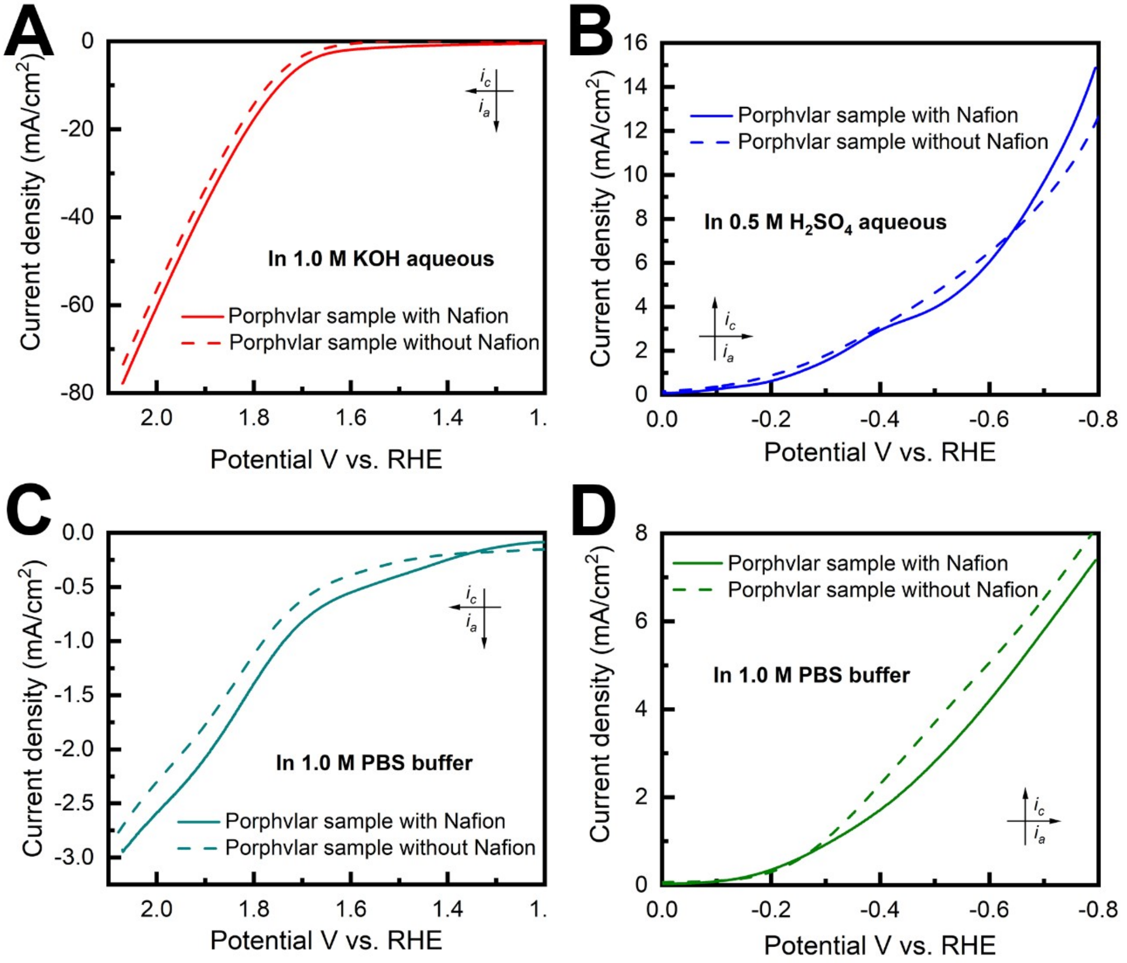

Fig. S14. Overlaid linear sweep voltammograms of Porphvlar sample with (solid line) and without (dashed line) Nafion® in the dropcast sample ink on carbon paper electrode: (A). OER in 1.0 M KOH aqueous solution; (B). HER in 0.5 M H_2SO_4 aqueous solution; (C). OER in 1.0 M PBS buffer; (D). HER in 1.0 M PBS buffer. The linear sweep voltammograms do not show large difference between with or without Nafion® sample. However, due to the smaller current density achieved when performing oxygen evolution reaction in 1.0 M PBS buffer, the current shifted but retained the overpotential.

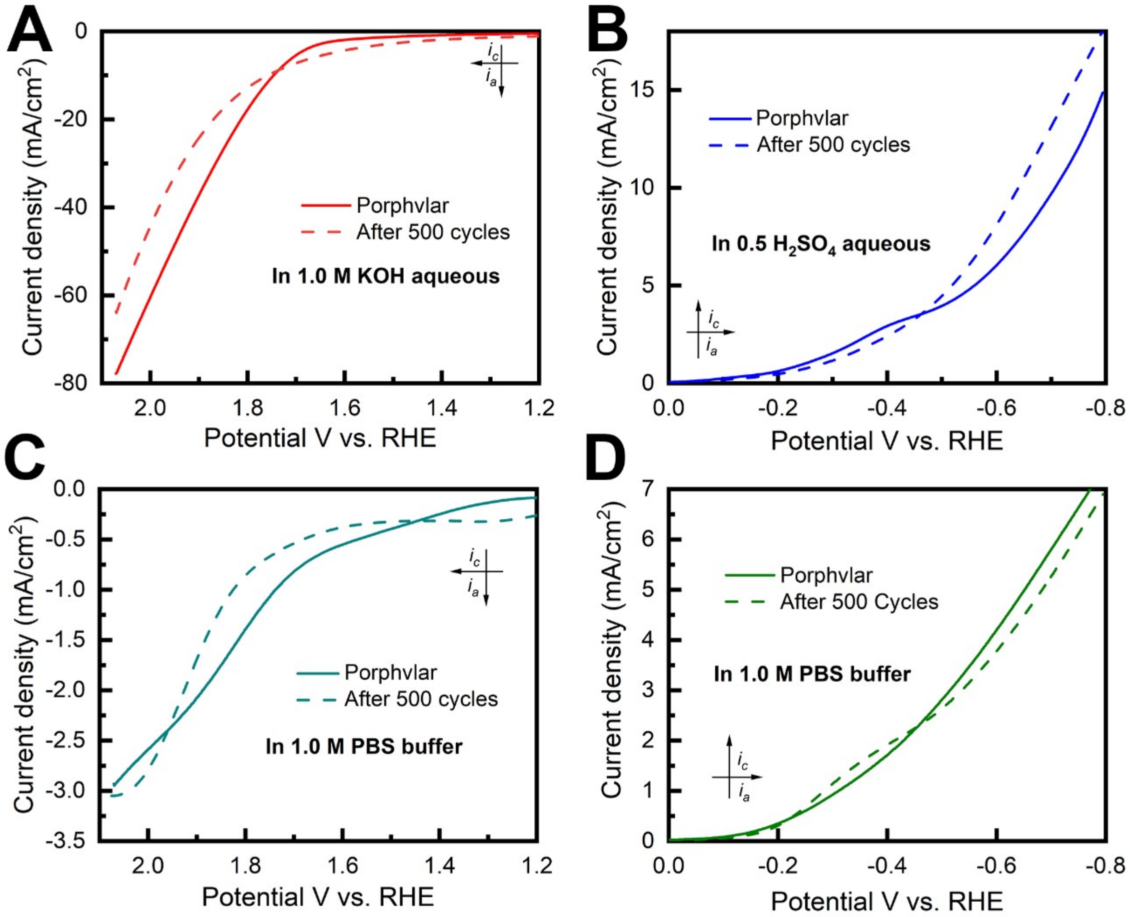

Fig. S15. Overlaid linear sweep voltammogram of Porphvlar before (dashed line) and after (solid line) 500 cycles on carbon paper electrode: (A). OER in 1.0 M KOH aqueous solution; (B). HER in 0.5 M H_2SO_4 aqueous solution; (C). OER in 1.0 M PBS buffer; (D). HER in 1.0 M PBS buffer. For HER, the catalytic overpotential retained after 500 cycle voltammetry in both acid and neutral electrolytes. For OER, the overpotentials shifted to more positive after 500 cycles.

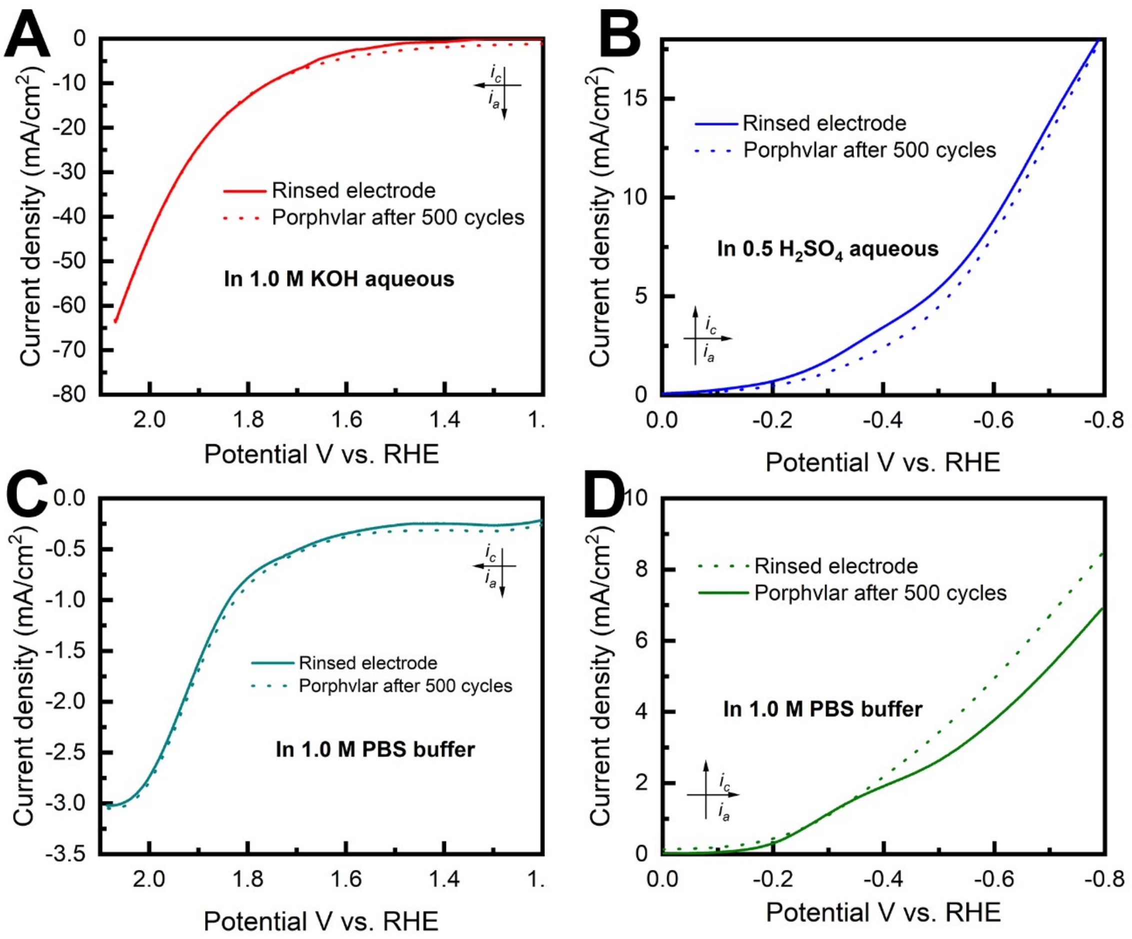

Fig. S16. Overlaid linear sweep voltammogram of the rinsed Porphvlar electrode (dashed line) and the Porphvlar electrode (solid line) after 500 cycles on carbon paper electrode: (A). OER in 1.0 M KOH aqueous solution; (B). HER in 0.5 M H_2SO_4 aqueous solution; (C). OER in 1.0 M PBS buffer; (D). HER in 1.0 M PBS buffer. No apparent overpotential shifts for Porphvlar working electrode under all the water splitting conditions, indicating no heterogeneous film formed during the 500 cycles voltammetry.

Electrochemical Active Surface Area (ECSA).

The electrochemical surface areas (ECSAs) are determined by measuring the double layer capacitance (C_{DL}) with different scan rates (from 0.02 v/s to 0.18 v/s) in a 0.1 V potential window centered around open circuit potential (OCP) which has no Faradaic reaction. The resulting capacitive current in the middle of the potential range is considered and is plotted as a function of scan rate that can result the double layer capacitance for the metal-free Porphvlar in different electrolytes (Figure S11). ECSAs are therefore calculated by the following equation:

$$ECSA = \frac{C_{DL}}{C_S}$$

where $C_S$is the specific capacitance of a certain material or the capacitance of an atomically smooth planar surface of the material per unit area under identical electrolyte condition, we adopt general specific capacitances of $C_S = 0.04\ mF/cm^2$ in 1.0 M KOH and $C_S = 0.035\ mF/cm^2$in 0.5 M H_2SO_4 according to the previously reported value.(3)

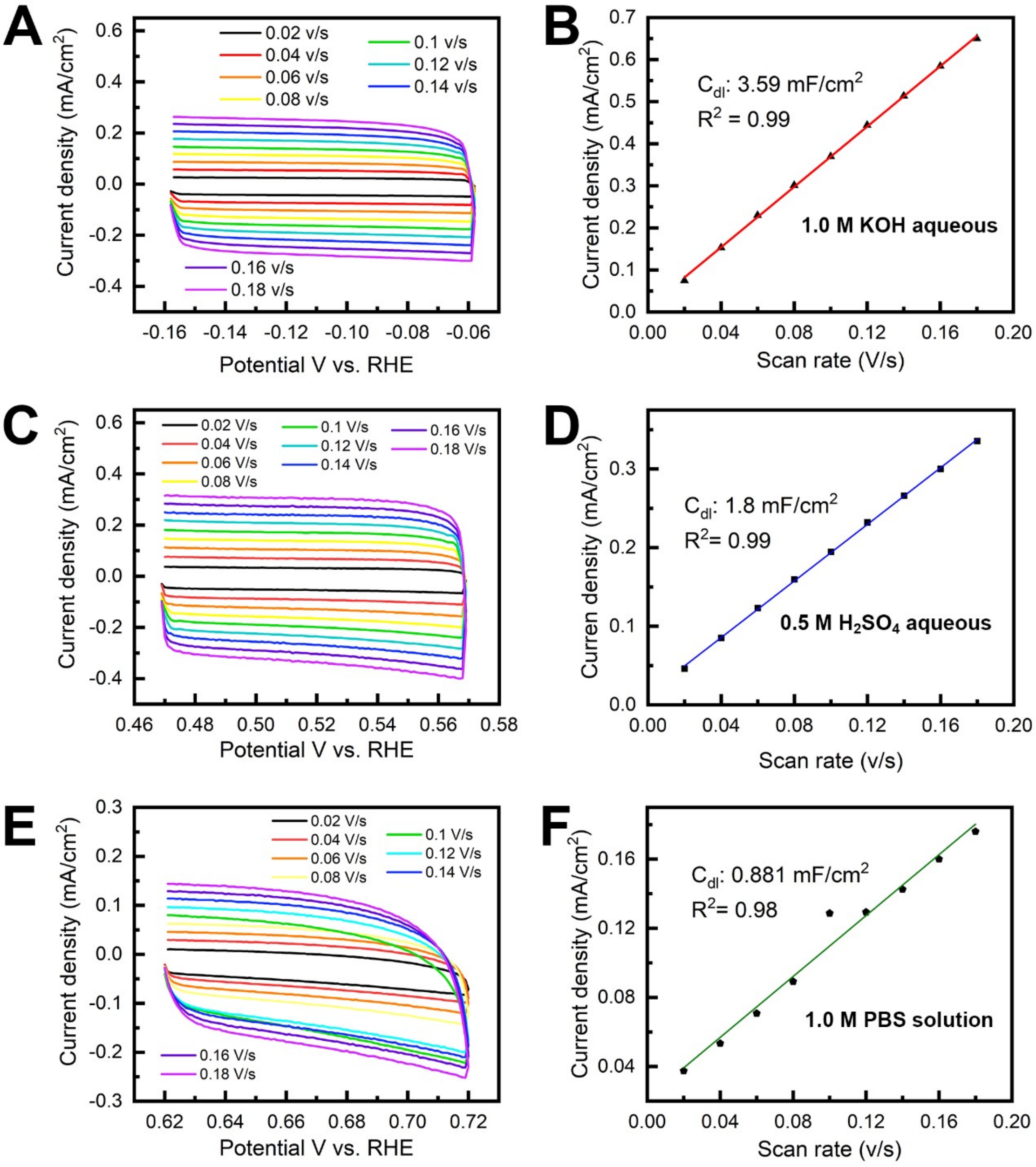

Fig. S17. Cyclic voltammograms of as-prepared Porphvlar at different scan rate in 0.1 V potential window with no faradaic process on carbon paper electrode: (A) in 1.0 M KOH aqueous solution; (C) in 0.5 M H_2SO_4 aqueous solution; (E) in 1.0 M PBS solution. Linear fit of scan rates as a function of double layer charging current for the Porphvlar report in this study: (B) in 1.0 M KOH aqueous, (D) in 0.5 M H_2SO_4 aqueous, (F) in 1.0 M PBS solution. Scan rate: 0.02 v/s, 0.04 v/s, 0.06 v/s, 0.06 v/s, 0.08 v/s, 0.1 v/s, 0.12 v/s, 0.14 v/s, 0.16 v/s, 0.18 v/s.

The Porphvlar material exhibit rectangle shape cyclic voltammogram in the non-faraday current region, indication good capacitor property of Porphvlar. The linear fit of current density as function of scan rate yields slope is the double layer capacitance value that generate from the interfaces between Porphvlar electrode and electrolytes. The larger the capacitance value the more electrochemical active surface area for the catalytic process. According to the equation (1), the $ECSA_{OER}$ in 1.0 M KOH aqueous solution is calculated to be 89.75 cm^2, whereas in 0.5 M H_2SO_4 solution, the $ECSA_{HER}$ is equal to 51.42 cm^2. Normalized the current density with respect to the electrochemical active surface area is shown in Figure S20.

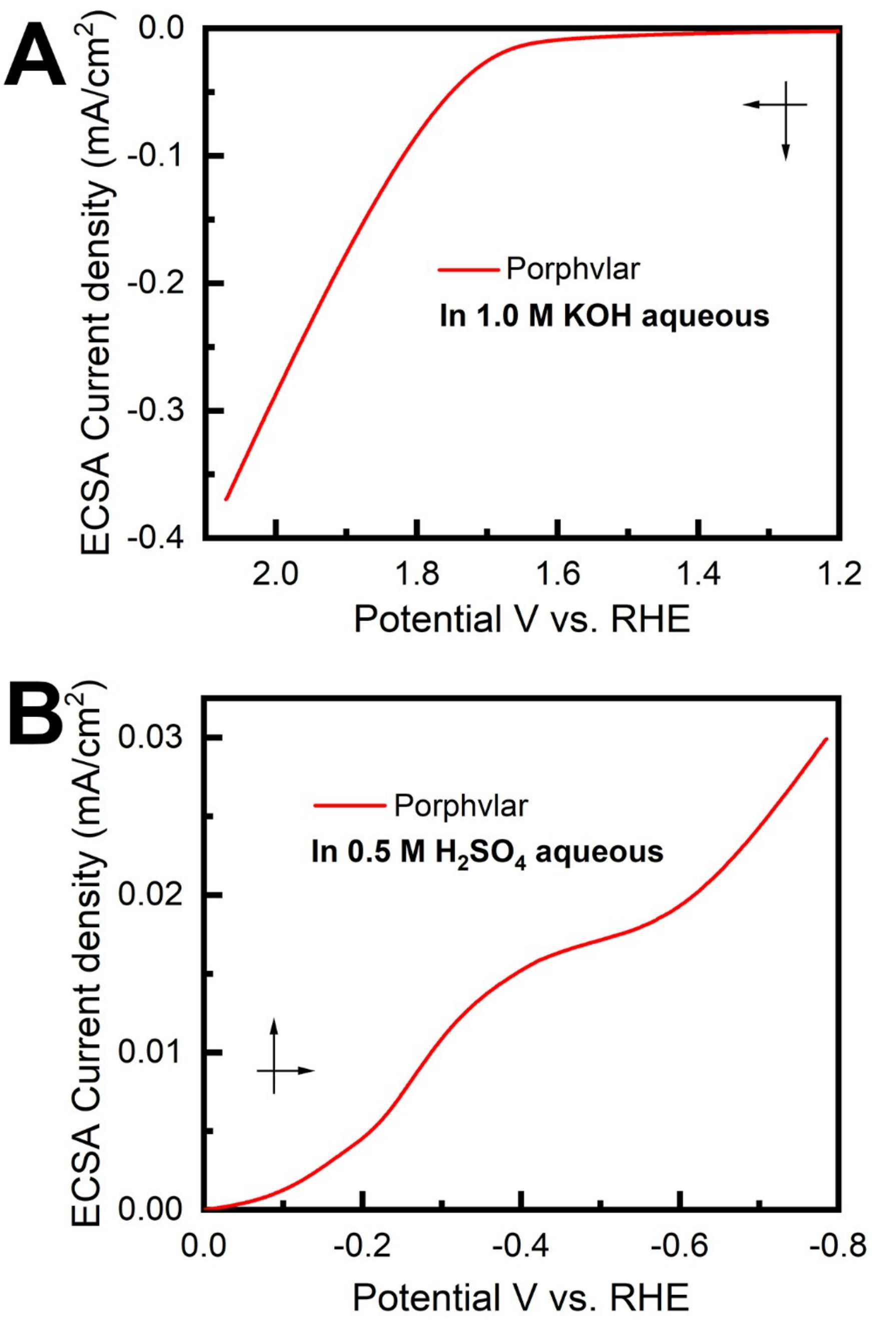

Fig. S18. Linear sweep voltammogram of Porphvlar with current density normalized with ECSA: (A) OER in 1.0 M KOH aqueous; (B) HER in 0.5 M H_2SO_4 aqueous.

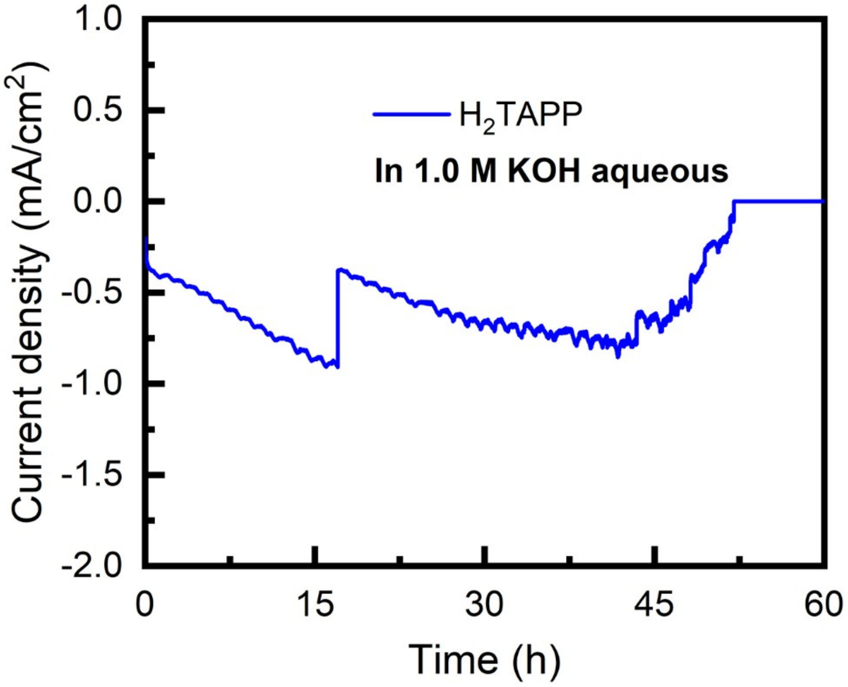

Fig. S19. Time dependence of the current density for molecular porphyrin (H_2TAPP) at static potential in 1.0 M KOH for 60 hours on carbon paper electrode. The current density varied throughout the bulk electrolysis and reached zero after 51 hours, which indicates the poor stability for molecular porphyrin as heterogeneous electrocatalyst for OER. For the generating hydrogen in 0.5 M H_2SO_4 aqueous solution, the protonation reaction occurred at amine core in H_2TAPP as soon as it immersed in the acidic electrolyte, which appears as the dropcasted H_2TAPP dissolved/reacted with the electrolyte with green color.

Synthesis of 5,10,15,20-tetrakis(4-aminophenyl) porphyrin (H_2TAPP)

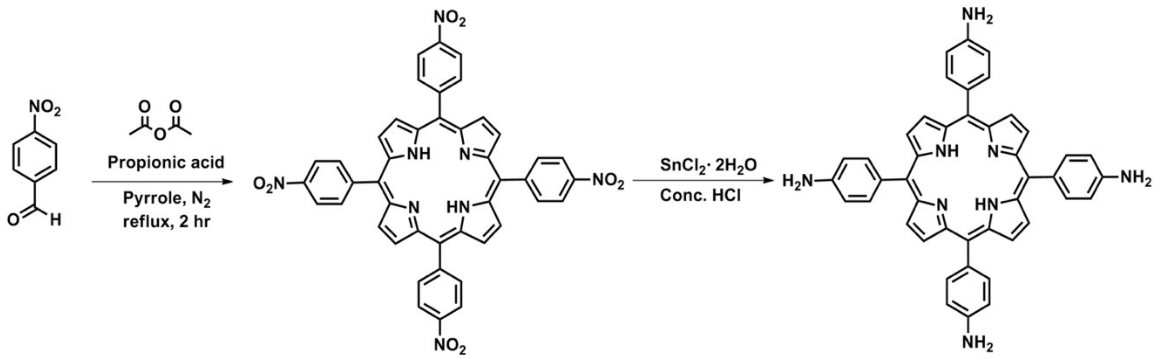

Scheme S1. Synthesis procedure of H_2TAPP.

5,10,15,20-tetrakis(4-aminophenyl) porphyrin (H_2TAPP) was synthesized by a modification of a previously reported procedure, depicted in Scheme S1.(4) A solution of 4-nitrobenzaldehyde (3.0 g, 20 mmol) and acetic anhydride (3.6 mL, 34.8 mmol) in 100 mL propionic acid was heated to 120°C. The mixture of freshly distilled pyrrole (1.4 mL, 20 mmol) was added slowly under N_2 and the reaction was stirred at 140°C for 2 h. Upon cooling, the mixture was refrigerated overnight, and then the resulting precipitate was collected by filtration and washed with MeOH and DI water. The obtained dark solid was dissolved in pyridine (20 mL) and refluxed for 1 h. After cooling down, the system was refrigerated overnight. The nitrated product 5,10,15,20-Tetrakis(4-nitrophenyl) porphyrin, H_2TNPP, was obtained by filtering. It was then washed with a 1:1 mixture of MeOH/acetone and dried under the vacuum yielding 3.7938g (4.76 mmol) of a purple solid. Without any further purification H_2TNPP was dissolved in 480 mL of concentrated hydrochloric acid. A solution of 27 g (130 mmol) $SnCl_2{\cdot}2H_2O$ in 120 mL concentrated hydrochloric acid was added to the porphyrin solution within 20 mins and was vigorously stirred for 3 h. Then the reaction mixture was placed in a hot water bath for 1.5 h. After this, the reaction was cooled down to room temperature, then put in an ice bath. The greenish solid obtained was dispersed in 1000 mL DI water. Concentrated ammonia was added dropwise in order to neutralize the excess acid until the pH of the solution was approximately 7. The purplish solid was washed twice with water, dried under vacuum at room temperature, and then Soxhlet-extracted with acetone for 24 h. The solvent was removed under reduced pressure to give isolated H_2TAPP as a purple crystal with a yield of 34.3% (1.1105g). UV-vis (λ_{max} nm in THF at 298 K), [log ε(103 $M^{-1}cm^{-1}$]: 429, 521, 570, 663. ^{1}H NMR (600 MHz, $CDCl_3$, 25°C): δ –2.72 (s, 2H, pyrrole –NH), 4.03 (s, 8H, amine –NH_2), 8.90 (s, 8H, β-pyrrole), 7.98 (d, 8H, J = 8.1 Hz, 2,6-(4-aminophenyl)), 7.07 (d, 8H, J = 8.1 Hz, 3,5-(4-aminophenyl)).

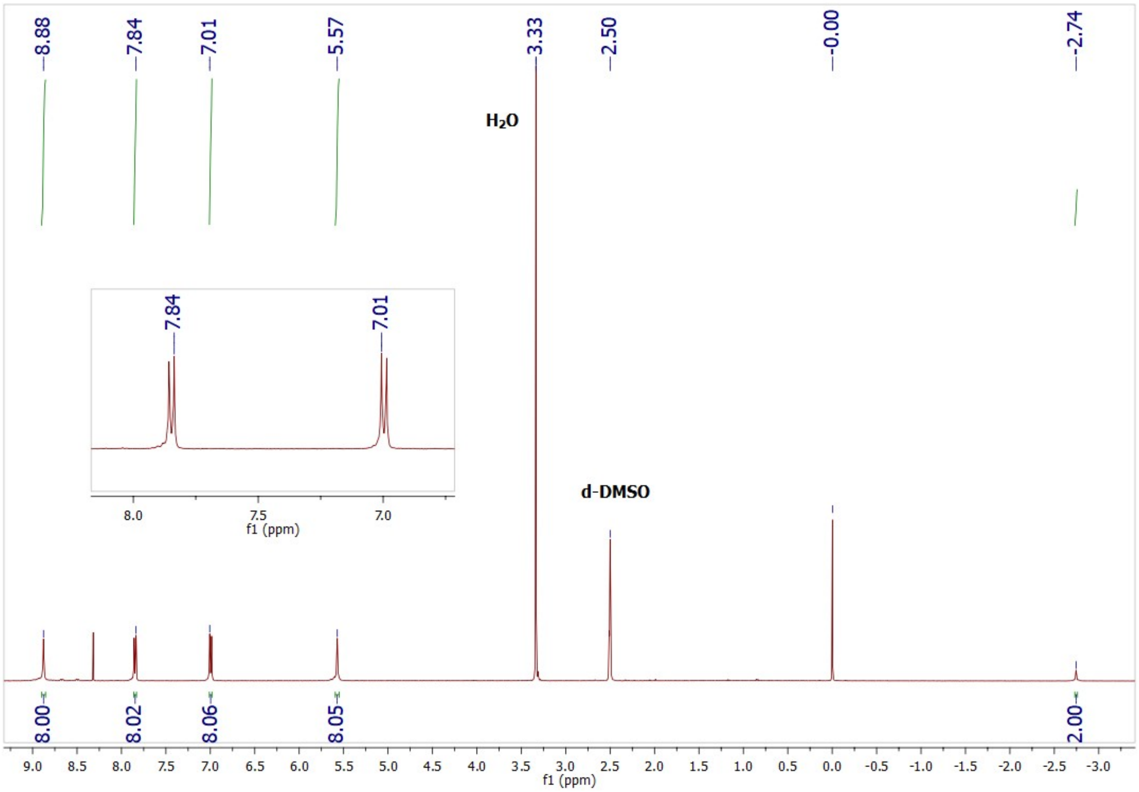

Fig. S20. ^{1}H NMR spectrum of 5,10,15,20-tetrakis(4-aminophenyl) porphyrin (H_2TAPP) in DMSO-D6.

Synthesis of Zinc(II) 5,10,15,20-tetrakis-(4-aminophenyl) porphyrin Zinc(II) (ZnTAPP)

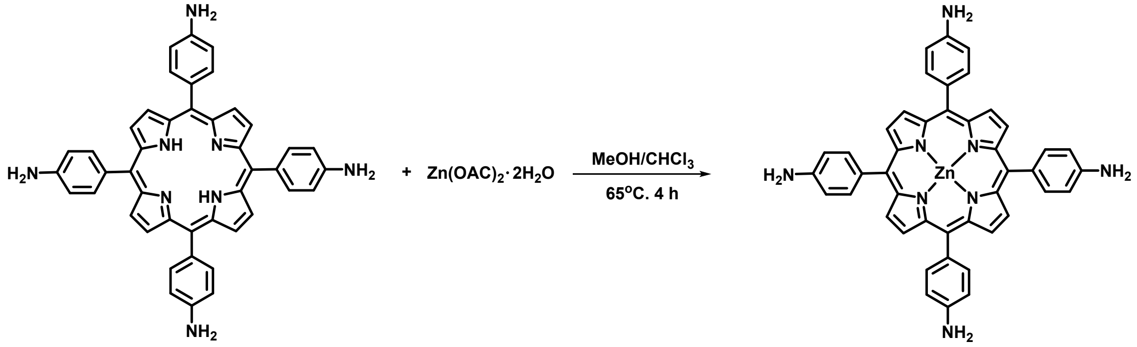

Scheme S2. Synthesis procedure of ZnTAPP.

H_2TAPP (1.0 mmol, 1.86 mg) and $Zn(OAc)_2 \cdot 2H_2O$ (4.0 mmol, 130 mg) were dissolved in a mixture solvent of methanol and chloroform under N_2 atmosphere. The mixture was refluxed for 4 hours at 65℃. After completion of reaction, the solvent was removed under reduced pressure and desired product was obtained by washing with DI water, followed by recrystallization in ethanol with a yield of 85.4%. UV-vis (λ_{max} nm in THF at 298 °K), [log ε (103 $M^{-1}cm^{-1}$]: 438, 561, 606. FT-IR (solid): 3349, 3222, 3227, 2316, 2109, 1591, 1505, 1401, 1276cm^{-1}

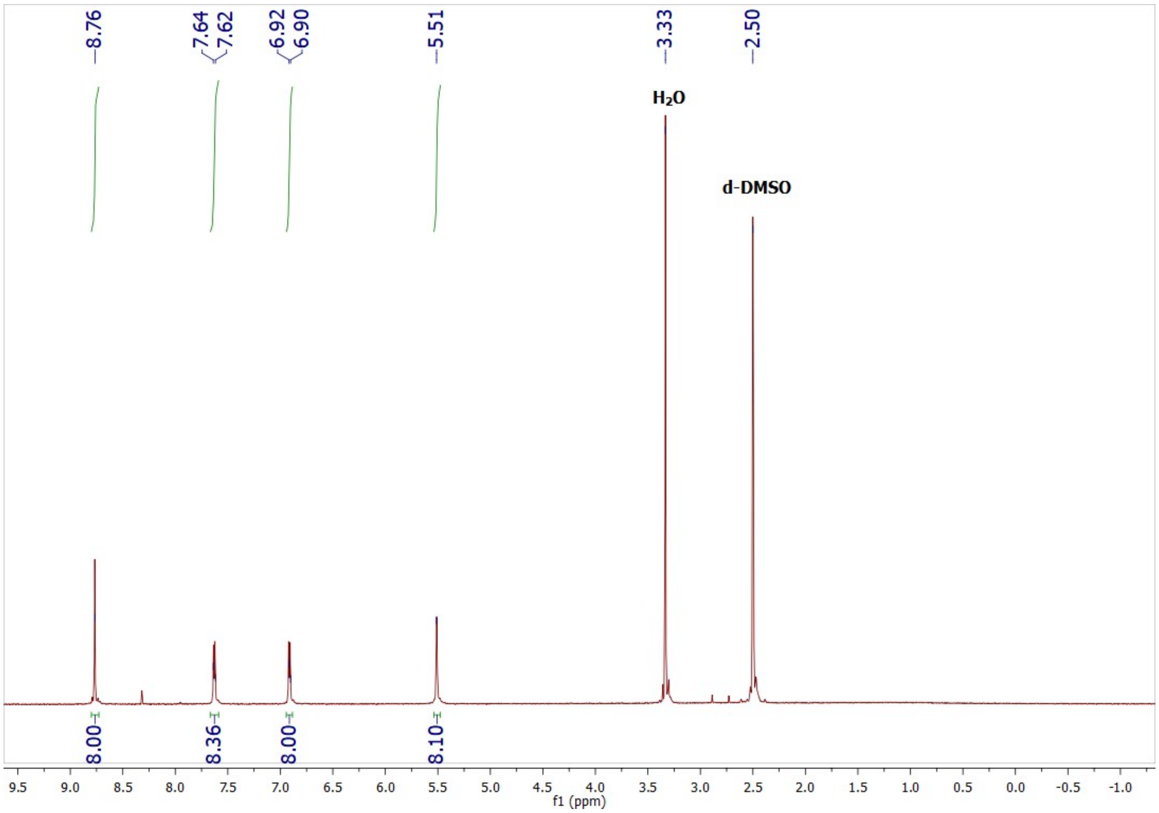

Fig. S21. ^{1}H NMR spectrum of Zn(II) 5,10,15,20-tetrakis(4-aminophenyl) porphyrin (ZnTAPP) in DMSO-D6.

Synthesis of Porphvlar

Porphvlar was synthesized through the polymerization of H_2TAPP and terephthaloyl chloride as modified from the synthesize method of the commercial PPTA threads, and is shown in Scheme 2 (main article).(5) Initially, N-methyl-2-pyrrolidone (NMP) 0.5 mL was added into 100 mL round bottom flask, and then bubbled N_2 to remove oxygen. When the temperature of the solvent reached 78°C, finely ground dry $CaCl_2$ was added and dissolved. H_2TAPP (72 mg, 0.106 mmol) and pyridine (0.025 mL) was added to the system with stirring. When H_2TAPP was completely dissolved, the temperature was lowered with an ice-bath. The polymerization was started by adding terephthaloyl chloride (88 mg, 0.425 mmol). After 1 h, DI water was added into the reaction and the resulting mixture was quickly filtered with a Buchner funnel and hydrophilic disks. The residue was washed with ethanol and water. The powder material was ground and kept immersed in ethanol overnight. The suspension was filtered again and dried under vacuum to yield a greenish powder (yield: 48.7 mg, 37.7%). UV-vis (λ_{max} nm in THF at 298°K), [log ε (103 $M^{-1}cm^{-1}$]: 425, 518, 556, 654. FT-IR (solid): 3000, 2960, 2378, 2126, 1681, 1587cm^{-1}.(5)

Synthesis of Zn-Porphvlar

Zn-Porphvlar was synthesized through the polymerization of ZnTAPP and terephthaloyl chloride with same method used in the Porphvlar synthesis. UV-vis (λ_{max} nm in THF at 298°K), [log ε (103 $M^{-1}cm^{-1}$]: 422, 514, 544, 601. FT-IR (solid): 2985, 2825, 2670, 2560, 2109,2077, 1986, 1679, 1430, 1273cm^{-1}

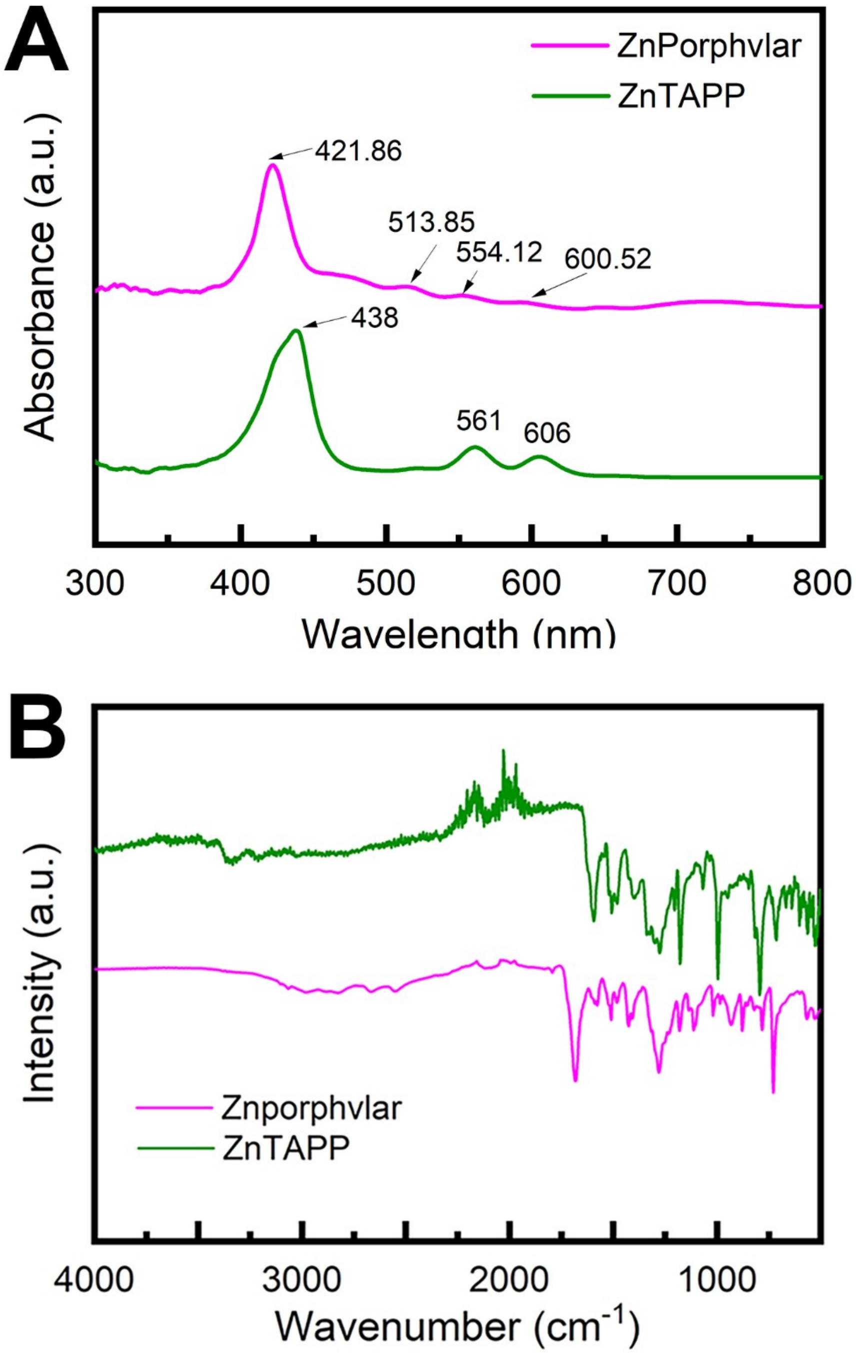

Fig. S22. Spectroscopy characterization of Zn-Porphvlar. (A) Comparison UV-vis spectra of Zn-Porphvlar and ZnTAPP; (B)FT-IR spectroscopy of Zn-Porphvlar and molecular porphyrin (ZnTAPP).

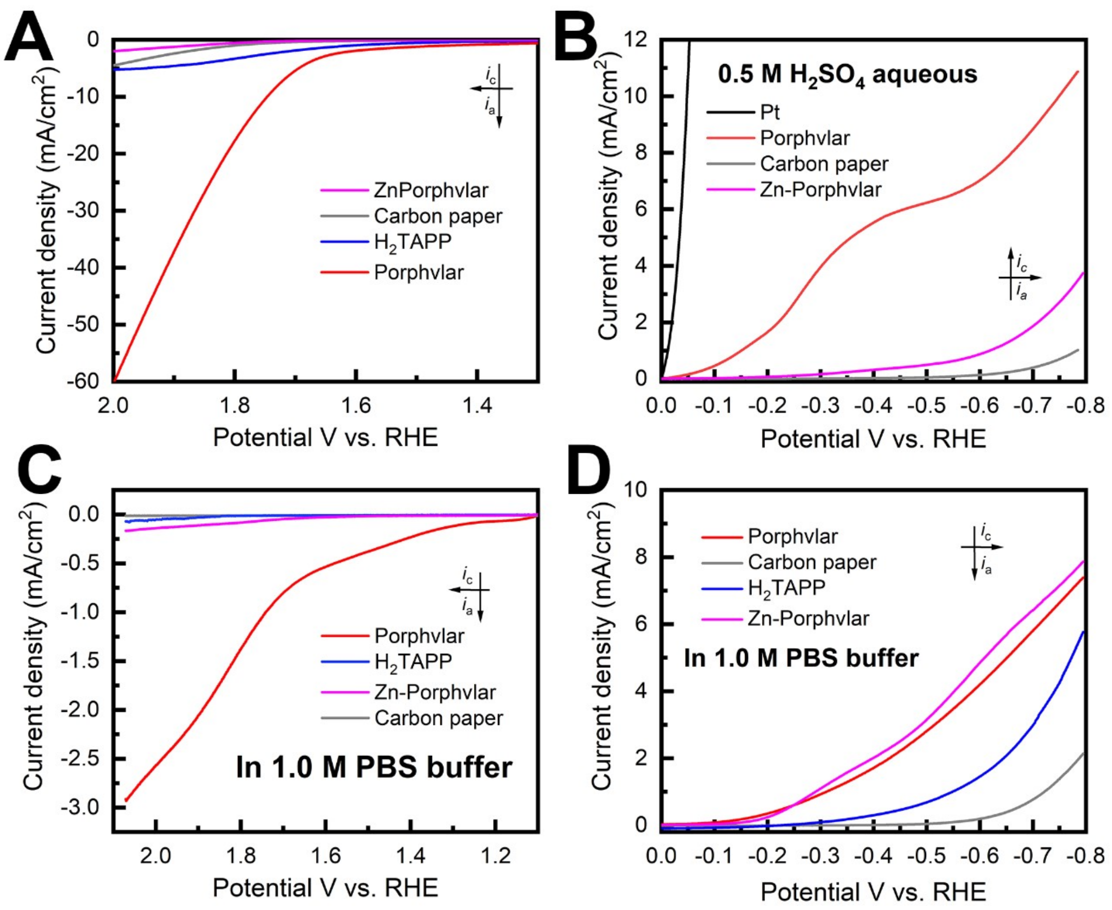

Fig. S23. Comparison linear sweep voltammetry of Zn-Porphvlar with the metal-free Porphvlar in different electrolytes under the corresponding oxidation and reductions on carbon paper electrode. Scan rate: 5 mV/s.

SI References

1. T. ul Haq, S. A. Mansour, A. Munir, Y. Haik, Gold-Supported Gadolinium Doped CoB Amorphous Sheet: A New Benchmark Electrocatalyst for Water Oxidation with High Turnover Frequency. *Advanced Functional Materials* **30**, 1910309 (2020).

2. J. Duan, S. Chen, C. Zhao, Ultrathin metal-organic framework array for efficient electrocatalytic water splitting. *Nat Commun* **8**, 15341 (2017).

3. C. C. L. McCrory, S. Jung, J. C. Peters, T. F. Jaramillo, Benchmarking Heterogeneous Electrocatalysts for the Oxygen Evolution Reaction. *J. Am. Chem. Soc.* **135**, 16977–16987 (2013).

4. G. Karimipour, S. Kowkabi, A. Naghiha, New aminoporphyrins bearing urea derivative substituents: synthesis, characterization, antibacterial and antifungal activity. *Brazilian Archives of Biology and Technology* **58**, 431–442 (2015).

5. H. Yan, *et al.*, A new approach to the preparation of poly(p -phenylene terephthalamide) nanofibers. *RSC Advances* **6**, 26599–26605 (2016).
